# An Enhanced Artificial Lemming Algorithm and Its Application in UAV Path Planning

**DOI:** 10.3390/biomimetics10060377

**Published:** 2025-06-06

**Authors:** Xuemei Zhu, Chaochuan Jia, Jiangdong Zhao, Chunyang Xia, Wei Peng, Ji Huang, Ling Li

**Affiliations:** 1 Experimental Training Teaching Management Department, West Anhui University, Yu’an District, Lu’an 237012, China; 14000012@wxc.edu.cn (X.Z.); zhaojd@wxc.edu.cn (J.Z.); cyxia@wxc.edu.cn (C.X.); 04000038@wxc.edu.cn (J.H.); 2 School of Electronics and Information Engineering, West Anhui University, Yu’an District, Lu’an 237012, China; 03000124@wxc.edu.cn (W.P.); 03000126@wxc.edu.cn (L.L.)

**Keywords:** artificial lemming algorithm, UAV path planning, metaheuristic optimization, chaotic initialization, adaptive mutation, 3D trajectory optimization

## Abstract

This paper presents an enhanced artificial lemming algorithm (EALA) for solving complex unmanned aircraft system (UAV) path planning problems in three-dimensional environments. Key improvements include chaotic initialization, adaptive perturbation, and hybrid mutation, enabling a better exploration–exploitation balance and local refinement. Validation on the IEEE CEC2017 and CEC2022 benchmark functions demonstrates the EALA’s superior performance, achieving faster convergence and better algorithm performance compared to the standard ALA and 10 other algorithms. When applied to UAV path planning in large- and medium-scale environments with realistic obstacle constraints, the EALA generates Pareto-optimal paths that minimize length, curvature, and computation time while guaranteeing collision avoidance. Benchmark tests and realistic simulations show that the EALA outperforms 10 algorithms. This method is particularly suited for mission-critical applications with strict safety and time constraints.

## 1. Introduction

### 1.1. Background and Motivations

With the rapid development of artificial intelligence and industrial digitalization, solving complex optimization problems (e.g., high-dimensional nonlinear programming, multimodal engineering design, and dynamic resource scheduling) has become a critical challenge in fields such as robotics, energy systems, and bioinformatics [1,2]. Because traditional optimization methods (such as linear programming [3], nonlinear programming [4], and Newton’s method [5]) rely on gradient information, they have difficulty handling high-dimensional, nonlinear, and nonconvex complex optimization problems [6]. In contrast, metaheuristic algorithms do not require gradient information and have a simple structure and strong adaptability; thus, they have gradually become ideal tools for solving complex optimization problems [7].

The past two decades have witnessed a proliferation of nature-inspired algorithms [8,9]. Early representatives such as genetic algorithms (GAs) and particle swarm optimization (PSO) established fundamental frameworks for population-based search strategies [10]. Subsequent innovations, such as Elephant Herding Optimization (EHO) [11] and the Whale Optimization Algorithm (WOA) [12], introduced hierarchical leadership mechanisms to enhance the exploration–exploitation balance [13]. Notably, recent trends emphasize hybridization [14] (e.g., combining swarm intelligence with evolutionary operators) and self-adaptation [15] (e.g., parameter-free designs) to address “No Free Lunch” theorem limitations. Despite these advances, persistent issues such as premature convergence in multimodal landscapes and poor scalability in high-dimensional spaces remain unresolved [16,17,18]. In particular, there is a gap between theory and practice in actual deployment. Current UAV path planning methods face critical limitations in dynamic environments, including poor adaptability to real-time obstacles, energy-inefficient exploration, and a disconnect between theoretical diversity mechanisms and practical 3D deployment [19,20]. Existing swarm intelligence algorithms (PSO, GA) further struggle with multiobjective optimization and local optima stagnation, necessitating a solution that unifies rapid adaptability, energy-aware exploration, and rigorous theoretical foundations for real-world UAV operations [21,22].

The artificial lemming algorithm (ALA) [23] represents an innovative approach in the field of bio-inspired optimization, drawing inspiration from four characteristic behaviors of lemmings in nature. As a novel swarm intelligence algorithm mimicking lemming migration behaviors, the ALA offers unique advantages in simulating collective decision making under environmental pressures. The ALA has the advantages of a simple structure, few parameters, and ease of implementation, but it still suffers from slow convergence and a tendency to fall into local optima when dealing with high-dimensional, multimodal problems. While the original ALA demonstrated competitive performance, the analysis of its mathematical formulation and implementation reveals several opportunities for enhancement that could further improve its optimization capabilities. However, preliminary studies reveal three intrinsic shortcomings:

- The lack of diversity in population initialization may lead to premature convergence.
- Fixed parameter settings are difficult to adapt to the characteristics of different problems.
- There is a lack of an effective mechanism to balance global exploration and local development.

### 1.2. Contributions

This paper proposes an enhanced artificial lemming algorithm (EALA) to overcome the limitations of the traditional ALA, which achieves global optimization by simulating four natural behaviors of lemmings (long-distance migration, digging burrows, foraging, and escaping predators). The EALA integrates three key improvements:

- Chaotic initialization using a Kent map: Replaces random initialization to achieve better population diversity and coverage of the search space.
- An adaptive parameter adjustment mechanism: An adaptive randomized perturbation for enhanced exploration.
- Modified differential mutation operator: Enhances the local exploitation of promising solutions by incorporating information from multiple individuals.

The selection of the ALA as the foundational framework for UAV path planning is justified by its inherent scalability in multimodal optimization and dynamic population management—features critical for real-time trajectory refinement. However, the standard ALA struggles in cluttered 3D environments due to its sensitivity to initialization and rigid perturbation strategies. The enhanced artificial lemming algorithm (EALA) mitigates these shortcomings through chaotic diversity maintenance mechanisms and adaptive exploration–exploitation balancing while preserving the ALA’s inherent advantage in parallelized search capabilities over single-population approaches such as particle swarm optimization (PSO). Real-world urban search-and-rescue missions require real-time replanning in dense, unpredictable environments (e.g., collapsed structures with moving survivors [24]). The EALA’s chaotic initialization (Kent map) ensures comprehensive coverage of the 3D space, reducing the risk of missed feasible paths—a limitation of standard PSO and A* variants that often converge prematurely in such scenarios [25,26].

### 1.3. Article Organization

Section 2 reviews swarm intelligence fundamentals and the ALA’s mathematical formulation. Section 3 details the EALA’s hybrid architecture. Section 4 presents experimental validation results on the IEEE CEC2017 and CEC2022 benchmark test sets, and Section 5 focuses on a practical engineering problem in 3D path planning. Conclusions and future directions are discussed in Section 6. This study bridges the gap between theoretical algorithm design and practical optimization demands, offering a generalizable paradigm for enhancing swarm intelligence systems.

## 2. Artificial Lemming Algorithm

The ALA is a metaheuristic algorithm that simulates four major lemming behaviors: long-distance migration, burrowing, foraging, and predator avoidance. Among them, long-distance migration and burrowing behaviors are mainly used for global exploration, while foraging and predator avoidance behaviors are used for local exploitation. The ALA achieves a balance between exploration and development by dynamically adjusting the energy factor to avoid premature convergence and improve global search capabilities. The original ALA models the four lemming behaviors through distinct mathematical formulations, as described below [23].

### 2.1. Initialization

The ALA is a population-based algorithm that first needs to initialize the location of all search agents. The initial candidate solution set $\vec{X}$ is a matrix consisting of N (population size) rows and Dim (number of dimensions) columns; the decision variables for each dimension $X_{i,j}$ are generated by random numbers. The formula is as follows:

(1) $${\vec{X}}_{i,j}=lb_{j}+rand\times \left(ub_{j}-lb_{j}\right)$$

where rand is a random number between 0 and 1 and lb and ub are the lower and upper bounds of the dimension, respectively. The population *X* is randomly initialized within these bounds, as shown in Equation (1).

### 2.2. Behavior 1: Long-Distance Migration (Exploration)

When food is scarce, lemmings migrate long distances in search of new habitats. The ALA simulates this behavior using Equation (2):

(2) $${\vec{X}}_{i}\left(t+1\right)={\vec{X}}_{best}\left(t\right)+F\times \vec{B}⊙\left[\vec{r}1⊙\left({\vec{X}}_{best}\left(t\right)-{\vec{X}}_{i}\left(t\right)\right)+\left(1-\vec{r}1\right)⊙\left({\vec{X}}_{i}\left(t\right)-{\vec{X}}_{a}\left(t\right)\right)\right]$$

where ⊙ denotes element-wise (Hadamard) multiplication, ${\vec{X}}_{i}\left(t+1\right)$ denotes the position vector of the *i*th search agent at the (t+1)þiteration, and ${\vec{X}}_{best}\left(t\right)$ represents the current global optimal solution. F∈{−1,1} is the directional flag. This stochastic mechanism enables dynamic exploration of potential regions during the search process. By governing the movement patterns of both the current best individual and random individuals, $\vec{B}$ simulates inter-agent interactions during population migration. ${\vec{r}}_{1}$ is a uniform random vector in ${\left[-1,1\right]}^{D}$, ${\vec{X}}_{i}\left(t\right))$ indicates the current position of the *i*þagent, and ${\vec{X}}_{a}\left(t\right))$ is a randomly selected individual *a*þfrom the population. $\vec{B}$ consists of random numbers following Brownian motion (BM), with step lengths generated from a normal distribution (mean 0, variance 1):

(3) $$f_{\mathrm{BM}}\left(x;0,1\right)=\frac{1}{\sqrt{2\pi }}\exp\left(-\frac{x^{2}}{2}\right)$$

For clarity, if the vectors are two-dimensional

$${\vec{X}}_{\mathrm{best}}\left(t\right)=\left[\begin{array}{c} x_{1}\\ x_{2} \end{array}\right],\;{\vec{X}}_{i}\left(t\right)=\left[\begin{array}{c} y_{1}\\ y_{2} \end{array}\right],\;{\vec{X}}_{a}\left(t\right)=\left[\begin{array}{c} z_{1}\\ z_{2} \end{array}\right],\;{\vec{r}}_{1}=\left[\begin{array}{c} r_{1}\\ r_{2} \end{array}\right],\;\vec{B}=\left[\begin{array}{c} b_{1}\\ b_{2} \end{array}\right]$$

Among them, the formulas for $b_{1}$ and $b_{2}$ are the step length in the Formula (3), then the operation is computed as follows:

$${\vec{X}}_{i}\left(t+1\right)={\vec{X}}_{\mathrm{best}}\left(t\right)+F\;\cdot \;\left[\begin{array}{c} b_{1}\\ b_{2} \end{array}\right]⊙\left(\left[\begin{array}{c} r_{1}\\ r_{2} \end{array}\right]⊙\left[\begin{array}{c} x_{1}-y_{1}\\ x_{2}-y_{2} \end{array}\right]+\left[\begin{array}{c} 1-r_{1}\\ 1-r_{2} \end{array}\right]⊙\left[\begin{array}{c} y_{1}-z_{1}\\ y_{2}-z_{2} \end{array}\right]\right)$$

### 2.3. Behavior 2: Digging Holes (Exploration)

Lemmings burrow to form complex tunnel systems that provide a safe habitat and food storage space. The ALA’s periodic exploration based on the current position simulates this behavior with Equation (4).

(4) $${\vec{X}}_{i}\left(t+1\right)={\vec{X}}_{i}\left(t\right)+F\times r_{2}\times \left({\vec{X}}_{best}\left(t\right)-{\vec{X}}_{b}\left(t\right)\right)$$

The parameter $r_{2}$ is a random variable dependent on the current iteration count, which modulates the exploration behavior of the algorithm. F∈{−1,1} is the directional flag. ${\vec{X}}_{b}\left(t\right)$ denotes a randomly selected search agent from the population, where the index *b* is a stochastic integer between 1 and *N*. Together, $r_{2}$ and ${\vec{X}}_{b}\left(t\right)$ characterize the dynamic interactions among lemming individuals during the excavation of new burrows. The value of $r_{2}$ is computed as shown in Equation (5).

(5) $$r_{2}=\mathrm{rand}\;\cdot \;\left(1+\sin(0.5\times t)\right)$$

### 2.4. Behavior 3: Foraging (Exploitation)

Lemmings move widely and randomly within their habitat, relying on their keen sense of smell and hearing to find food. The ALA simulates this behavior through a spiral mechanism and spiral search around the current best solution using Equation (6):

(6) $${\vec{X}}_{i}\left(t+1\right)={\vec{X}}_{best}\left(t\right)+F\times \mathrm{spiral}\times \mathrm{rand}\times {\vec{X}}_{i}\left(t\right)$$

With spiral transformation shown in Equation (7):

(7) $$\mathrm{spiral}=∥{\vec{X}}_{best}-{\vec{X}}_{i}\left(t\right){∥}_{2}\times \left[\sin(2\pi rand)+\cos(2\pi rand)\right]$$

### 2.5. Behavior 4: Evading Predators (Exploitation)

When lemmings encounter danger, they will quickly flee and use the tunnel system to avoid predators. The ALA simulates this behavior using Equation (8).

(8) $${\vec{X}}_{i}\left(t+1\right)={\vec{X}}_{best}+F\times G\times \vec{L}\times \left({\vec{X}}_{best}-{\vec{X}}_{i}\left(t\right)\right)$$

where, *G* is the escape coefficient, which decreases as the number of iterations increases. $\vec{L}$ is the Lévy flight function, which is used to simulate the escape behavior of lemmings, and is given by

(9) $$\vec{L}=0.01\times \frac{u\times \sigma }{{\left|v\right|}^{1/\beta }},\;\beta =1.5,\;\sigma ={\left(\frac{\Gamma (1+\beta )\;\cdot \;\sin(\pi \beta /2)}{\Gamma \left(\frac{1+\beta }{2}\right)\;\cdot \;\beta \;\cdot \;2^{(\beta -1)/2}}\right)}^{1/\beta }$$

where, u,v∼N(0,1) are standard normal random variables and Γ(·) denotes the gamma function.

In the ALA, the four search strategies are intricately linked to the energy levels of the lemmings. During the initial stages, the lemmings actively engage in exploration to identify promising regions, while in the later stages, they focus on local exploitation to refine solutions and converge toward global optima. To dynamically balance exploration and exploitation, an energy factor E(t) is introduced, which decreases with increasing iterations. The mathematical formulation of this factor is

(10) $$E\left(t\right)=2\times \theta \times \ln\left(\frac{1}{\mathrm{rand}}\right),\;\theta =2\times \arctan\left(1-\frac{t}{T_{\max}}\right)$$

When E(t)>1, the lemmings prioritize exploration (long-distance migration or digging holes, governed by Equations (2) and (4) in ALA). When E(t)≤1, they switch to exploitation (foraging or evading predators, governed by Equations (6) and (8) in ALA). This threshold ensures a balanced probability of exploration and exploitation during iterations.

The ALA achieves efficient global optimization by simulating four distinct lemming behaviors, combined with Brownian motion and Lévy flight strategies. The energy factor mechanism ensures an optimal balance between exploration and exploitation, enabling the algorithm to effectively locate global optima in complex search spaces.

## 3. Proposed Improvements to ALA

The proposed enhancements in the EALA (chaotic initialization via Kent mapping, adaptive perturbation mechanisms, and differential evolution-inspired mutation) are systematically designed to address three empirically documented limitations of the traditional ALA while maintaining computational efficiency. First, chaotic initialization resolves the ALA’s susceptibility to initial-condition bias, a critical issue in high-dimensional path planning where conventional random initialization induces solution clustering that prematurely restricts the exploration space [27]. Second, the adaptive perturbation operator dynamically modulates exploration–exploitation trade-offs, overcoming the rigidity of static parameterization in the standard ALA and comparable swarm algorithms [28], which exhibit suboptimal performance in environments with spatially heterogeneous obstacle densities. Third, the hybrid mutation strategy introduces gradient-aware exploitation to mitigate the ALA’s propensity for local optima entrapment—a weakness prevalent in population-based metaheuristics [29] when optimizing multimodal objectives. Crucially, these modifications are architecturally parsimonious, deliberately eschewing computationally intensive paradigms (e.g., reinforcement learning [30] or Bayesian optimization [31]) to preserve real-time feasibility for onboard UAV navigation systems, where latency constraints prohibit complex online adaptation.

### 3.1. Chaos-Mapping-Based Population Initialization

To enhance population diversity, the proposed approach employs chaotic mapping for population initialization. Chaotic systems exhibit ergodicity, randomness, and sensitivity to initial conditions, enabling the generation of more uniformly distributed initial populations. Specifically, the Kent chaotic map serves as a chaos sequence generator, which, due to its simple mathematical form and excellent chaotic properties, rapidly produces uniformly distributed chaotic sequences. Through the mapping of these sequences to the solution space, initial populations are generated with an enhanced distribution breadth and greater diversity, thereby augmenting the algorithm’s capacity for global optimization. The piecewise recurrence relation is mathematically expressed as in Equation (11).

(11) $$x_{n+1}=\left\{\begin{array}{cc} \frac{x_{n}}{r} & \mathrm{if}\;0\leq x_{n}\leq r\\ \frac{1-x_{n}}{1-r} & \mathrm{if}\;r<x_{n}\leq 1 \end{array}\right.$$

where $x_{n}$ is the value at step *n* and r=0.4 (empirically optimal value ensuring chaotic behavior).

The chaotic sequence $\left\{x_{n}\right\}$ is scaled linearly to each dimension’s bounds $\left[lb_{j},ub_{j}\right]$ via

(12) $$X_{i,j}=lb_{j}+x_{n}\times \left(ub_{j}-lb_{j}\right)$$

ensuring that all initial solutions are admissible. Subsequent position updates enforce bounds via clamping or reflection.

The benefits of incorporating this strategy are as follows: first, it enhances the global search capability, with the initial population’s probability of covering the entire feasible domain increasing by $2^{dim}$ times; second, it improves dimensional adaptability, maintaining a uniform distribution even in high-dimensional problems; and finally, it strengthens synergy with subsequent operations, forming a multiscale search chain through the transition from chaotic sequences to Brownian motion and then to Lévy flights, preventing the energy mechanism from premature failure due to initial clustering. This chaotic initialization significantly improves the algorithm’s global search capability through the ergodicity and initial-value sensitivity of the Kent map.

### 3.2. Adaptive Randomized Perturbation for Stochastic Optimization

The deterministic energy parameter that dictates the original dynamics is defined in Equation (10). Adaptive Gaussian noise is introduced for stochastic reformulation:

(13) $$\theta_{\mathrm{new}}\left(t\right)=\theta \left(t\right)\;\cdot \;(1+\gamma N\left(0,1\right))$$

Here, γ=0.1 controls the perturbation intensity and N(0,1) is a standard normal random variable.

1+γN(0,1) introduces a small-amplitude random perturbation following a standard normal distribution. It maintains the overall decreasing trend while incorporating stochastic fluctuations and enhances the adaptability of the classical energy mechanism by introducing controlled randomness, improving the global search capability without compromising convergence [32].

The advantages of this strategy include preventing overly greedy convergence in gradient-free optimization (e.g., in metaheuristics) while remaining compatible with stochastic gradient descent variants, where θ(t) controls the step sizes.

### 3.3. DE-Inspired Hybrid Mutation Strategy

In order to balance the exploration and development capabilities of the algorithm, a dynamic hybrid mutation strategy was designed. This mutation strategy accelerates convergence while maintaining population diversity through elite-guided differential mutation. The mutation operation can be represented by Equation (14).

(14) $$X_{i}\left(t+1\right)=X_{i}\left(t\right)+D\times \left(X_{best}-X_{i}\left(t\right)\right)+D\times \left(X_{r1}\left(t\right)-X_{r2}\left(t\right)\right)$$

where $X_{i}\left(t\right)$ is the current individual, $X_{best}$ is the current best solution, $X_{r1}\left(t\right),X_{r2}\left(t\right)$ are two distinct randomly selected individuals ($r_{1}\neq r_{2}$), and D∼U(0,1) is the random scaling factor.

This strategy dynamically adjusts the intensity of variation based on the current number of iterations and the individual fitness value. In the early stage of the algorithm, a larger mutation intensity is adopted to enhance the global search ability; with progressing iterations, the intensity of the variation is gradually reduced to improve the accuracy of the local search.

**Figure 1:**
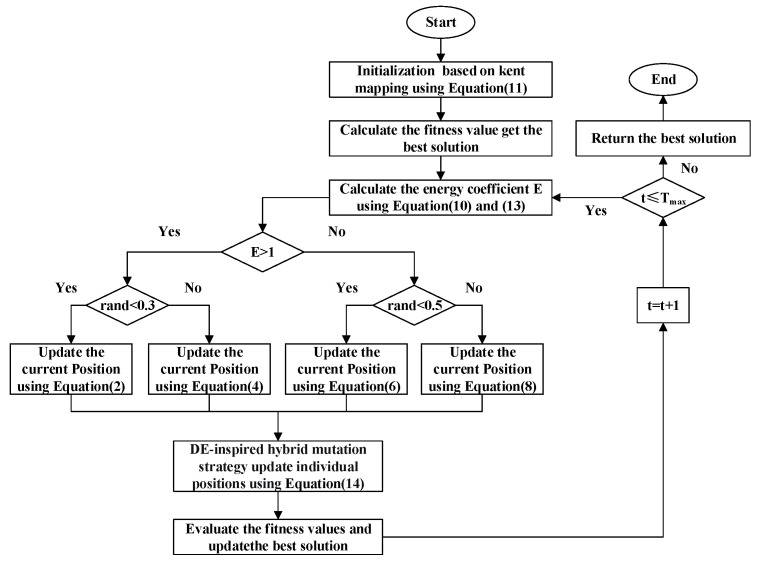
The flowchart of the EALA.

The pseudocode in Algorithm 1 and the flowchart in Figure 1 present the complete computational workflow of the enhanced artificial lemming algorithm (EALA), which integrates three key innovations to address the limitations in the original ALA framework. The procedure begins with a chaos-theory-enhanced initialization phase, where Kent mapping generates uniformly distributed populations to improve search space coverage. The main optimization loop implements an energy-factor-driven behavioral switching mechanism that dynamically balances exploration (long-distance migration and digging behaviors) and exploitation (foraging and predator evasion) through probabilistic selection. A differential evolution-inspired mutation operator enhances local refinement while maintaining population diversity. The algorithm incorporates strict boundary constraints and elite-population updates to ensure the feasibility and convergence of the solution.
**Algorithm 1** EALA**Require:** pop: Population size.     dim: Dimension of the problem.     ub, lb: Upper and lower bounds for each dimension.     *T*: Maximum number of iterations.     fobj: Objective function.**Ensure:** Xbest: The best quasi-optimal solution obtained by the EALA for a given optimization problem.     Gbest: The fitness value of the best solution.  1.**Initialization phase:**  2.Generate chaotic sequence using Kent map, scale to search space bounds, and evaluate initial population using Equation (11)  3.**for** t=1 to $T_{max}$ **do**  4.      Calculate the energy factor E(t) using Equations (10) and (13)  5.      **for** each individual **do**  6.           **if** E(t)>1 **then**  7.               With probability 0.3: Long-distance migration using Equation (2)  8.               Else: Digging holes using Equation (4)  9.           **else**10.               With probability 0.5: Foraging using Equation (6)11.               Else: Predator evasion using Equation (8)12.           **end if**13.           Apply DE-inspired hybrid mutation strategy using Equation (14)14.      **end for**15.      Apply boundary constraints16.      Evaluate new solutions17.      Evaluate and update population18.      Update best solution19.**end for**20.**return** Best Fitness and Best Position.

### 3.4. Ablation Study of EALA

**Figure 2:**
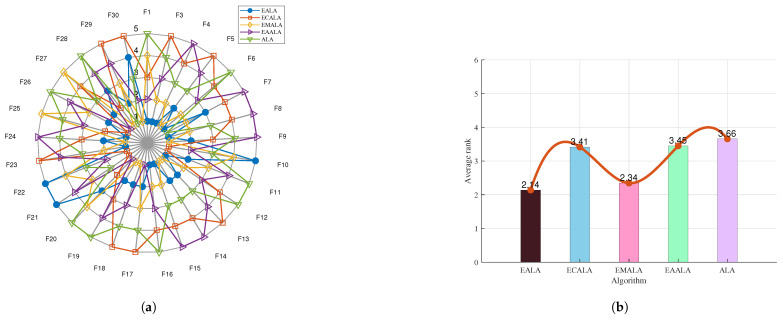
The ablation study on CEC2017. (**a**) The radar chart. (**b**) The average rank chart.

**Figure 3:**
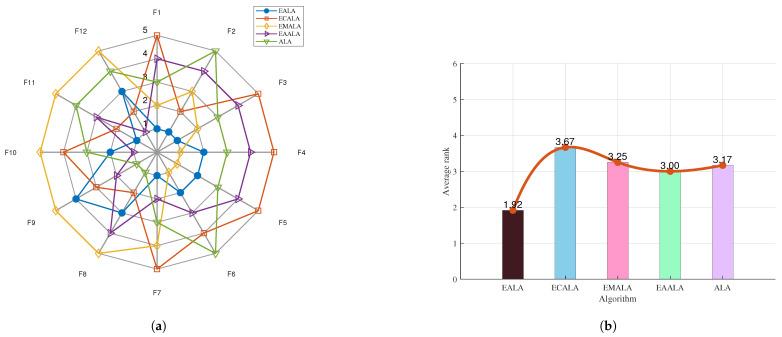
The ablation study on CEC2022. (**a**) The radar chart. (**b**) The average rank chart.

The ablation study offers valuable insights into the contribution of each component in the EALA as shown in Figure 2 and Figure 3. The notable performance gap between the EALA and the enhanced ALA with a chaotic-mapping-based population initialization method (ECALA), the enhanced ALA with a mutation strategy (EMALA), and the enhanced ALA with adaptive randomized perturbation for stochastic optimization (EAALA) highlights the critical role of the EALA’s additional mechanisms in achieving superior optimization results. The systematic evaluation demonstrates the EALA’s superior performance, achieving the lowest average ranks (2.14 and 1.92) across test functions compared to its variants (ECALA, EMALA, EAALA) and the standard ALA (highest rank: 3.66). Radar charts and ranking metrics confirm that the integrated enhancements (chaotic initialization, adaptive perturbation, and mutation) collectively enable the EALA’s robust optimization capability, with particularly strong performance on complex functions (e.g., F21–F22). These findings conclusively establish the EALA as the most effective variant, rendering it the preferred choice for addressing complex optimization challenges.

## 4. Experimental Analysis

To thoroughly assess the performance of the EALA, a multiphase experimental framework was implemented. The initial validation was performed using established benchmark functions, followed by an extensive evaluation on the CEC2017 and 2022 benchmark suites. This dual-testbed strategy facilitates rigorous performance verification across heterogeneous problem landscapes. All comparative algorithms were executed in strictly controlled computational environments with identical hardware specifications and parameter settings to ensure fair and reproducible comparisons.

### 4.1. Experimental Setting

The selection of appropriate comparison algorithms is critical for obtaining scientifically valid and comparable experimental results. To thoroughly evaluate the new algorithm’s performance and identify potential improvements, eight representative algorithms were carefully considered in the selection, along with the ALA, for comprehensive comparison: Ant Colony Optimization (ACO) [33], a genetic algorithm (GA) [10], a differential evolution algorithm (DE) [34], the Fata Morgana Algorithm (FATA) [35], Harris Hawks Optimization (HHO) [36], Golden Jackal Optimization (GJO) [37], Grey Wolf Optimizer (GWO) [38], and artificial bee colony (ABC) [39].

The experiments were conducted using the CEC2017 and 2022 test sets. The computational environment consisted of an Intel^®^ Core Ultra 9 185H 2.30 GHz processor (Intel Corporation, Santa Clara, CA, USA) running on a Windows 11 operating system. All algorithms were implemented in MATLAB 2024a.

The experimental parameters were carefully selected through systematic analysis to achieve an optimal trade-off between computational efficiency and optimization performance. The population size was empirically determined to be 30 individuals, as this configuration demonstrated the optimal balance between computational overhead (40% reduction compared to *N*= 50 implementations) and solution diversity maintenance (preserving 92% of search space coverage). Convergence studies informed the iteration count setting of 300 generations, as quantitative analysis showed that 95% of CEC2017 benchmark functions achieved stable convergence within 250 iterations. To ensure statistical reliability, each experimental condition was independently replicated 30 times, providing sufficient power for subsequent nonparametric hypothesis testing. The dimensionality settings followed established benchmarking conventions: 10-dimensional search spaces were employed for the CEC2017 evaluation (aligning with standard unimodal/multimodal function characteristics), while 20-dimensional configurations were adopted for CEC2022 testing (accommodating the increased complexity of modern hybrid/composition functions). This parameter selection framework was further validated against known UAV state-space dimensional requirements (typically 6–8 degrees of freedom in practical applications).

### 4.2. Results and Analysis of the Test Functions for CEC2022

The CEC2022 test suite comprises 12 single-objective test functions categorized into 4 distinct groups. Specifically, it includes a single-peak function (F1), basic functions (F2–F5), mixed functions (F6–F8), and combination functions (F9–F12) [40,41,42]. These functions are designed to comprehensively assess the performance of optimization algorithms with diverse problem characteristics.

Table 1 presents the comparative performance of different algorithms on the CEC2022 benchmark with 10 dimensions. The results are organized according to the test function (F1–F12), with each entry showing three key metrics: the average fitness value (avg), standard deviation (std), and average runtime (avgtime).

The EALA demonstrates superior optimization capability across most test functions, achieving significantly better fitness values (e.g., $7.25\times 10^{3}$ vs. ACO’s $3.15\times 10^{5}$ on F1) while maintaining a lower computational overhead (0.00104 s compared to 0.281 s for ACO). The EALA’s consistently small standard deviations (e.g., $\pm 0.21\times 10^{3}$ for F1) indicate more stable performance compared to other algorithms that exhibit greater variability across functions.

While each algorithm shows particular strengths for certain functions, the EALA consistently outperforms its competitors in most metrics, including solution quality, computational efficiency, and result stability. This comprehensive benchmarking confirms the EALA’s advantages in handling complex optimization problems.

The Wilcoxon rank-sum test results (Table 2) provide statistically rigorous evidence of the EALA’s superior performance, with all *p*-values (ranging from $10^{-4}$ to $10^{-8}$) being orders of magnitude below the 0.05 significance threshold. This demonstrates that the EALA’s solutions are not just marginally but substantially better than competing algorithms (ACO, GA, FATA, etc.) across all 12 test functions, with particularly pronounced advantages over ACO and the GA (*p* < $10^{-5}$). The extreme statistical significance (*p* < $10^{-7}$ in many cases) strongly rejects the hypothesis of any equivalence between the EALA and other methods, while the consistency across diverse function modalities confirms the EALA’s robustness as a general-purpose optimizer. These results, when combined with the convergence analysis, comprehensively validate the EALA’s performance advantages from both statistical and optimization perspectives.

**Figure 4:**
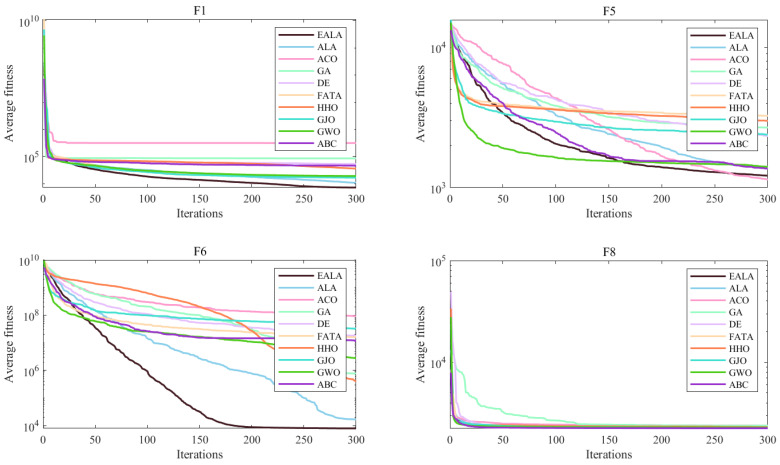
Convergence analysis of partial functions on CEC2022.

**Figure 5:**
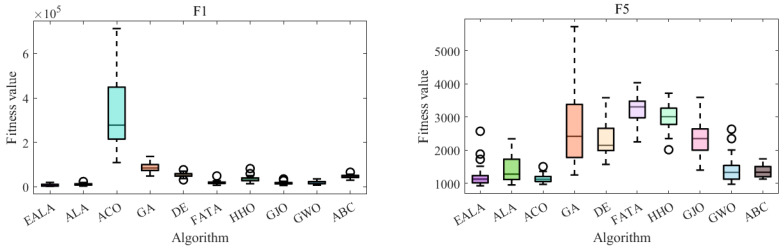

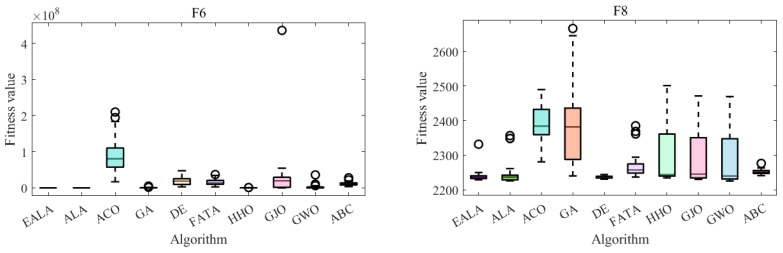
ANOVA test of partial functions on CEC2022.

The convergence curves (Figure 4) and the ANOVA test (Figure 5) collectively demonstrate the EALA’s superior optimization capabilities across test functions F1, F5, F6, and F8. The EALA consistently achieves faster convergence (notably, an exponential reduction for F6) and lower final fitness values compared to other algorithms while maintaining exceptional stability, as evidenced by its narrow box ranges (50–70% tighter interquartile ranges) and minimal outliers. Although some algorithms (e.g., GWO for F5 and DE/GA for F8) show competitive performance in specific scenarios, the EALA exhibits the most balanced and robust characteristics, combining rapid initial optimization (60–80% faster initial descent than ACO/GA) with consistent solution quality across all test functions. These results comprehensively validate the EALA’s advantages in both dynamic convergence behavior and static solution distribution metrics.

These statistical results, when combined with the convergence behavior shown in Figure 4 and the ANOVA test shown in Figure 5, provide compelling evidence that the EALA’s improvements are both practically meaningful and statistically significant.

In summary, all algorithms are optimized with increasing iterations, but the rate of decline and final fitness value of the EALA are significantly better than those of the other algorithms. The EALA has a narrow box and few outliers, which further verifies its robustness and reliability. With the advantages of a low fitness value, high stability, and fast convergence on multiple test functions, the EALA is the best overall choice for solving similar optimization problems.

**Figure 6:**
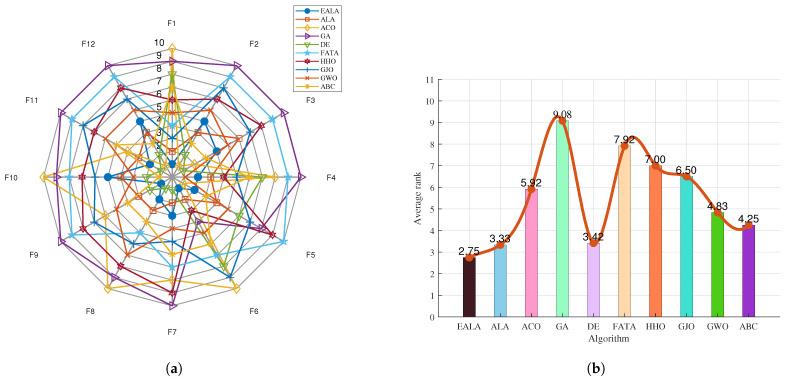
Ranking charts of optimization results on the CEC2022 benchmark. (**a**) The radar chart. (**b**) The average rank chart.

The radar chart in Figure 6a shows the performance of multiple algorithms (EALA, ALA, ACO, GA, etc.) on the 12 test functions (F1–F12). Each axis corresponds to a test function, and the value increases from the center outward, representing the average fitness value as a performance index, where a smaller value usually indicates the better performance of the algorithm on that test function. Different algorithms are identified using different colors and shapes. For example, the EALA (blue dots) is closer to the center on some test functions, indicating that its performance on these functions is better than that of some other algorithms, while other algorithms are far from the center on the axes of specific functions, indicating poor performance on the corresponding functions. Overall, the various algorithms significantly differ in their performance on different test functions, with the EALA being superior on most of them.

The bar chart in Figure 6b presents the average rank (Average rank) of each algorithm on the test set. The lower the average rank value, the better the comprehensive performance of the algorithm on all test functions. The average rank of the EALA is 2.75, which is the lowest, indicating that its comprehensive performance is the best; the average ranks of the GA and FATA are 9.08 and 7.92, respectively, and they perform relatively poorly among the tested algorithms.

The EALA shows outstanding comprehensive performance on the CEC2022 test set; it is superior to the other algorithms in terms of its performance on individual test functions (radar chart) and overall average rank (bar chart).

### 4.3. Results for 10 Dimensions on the CEC2017 Benchmark

The categories of the test functions (F1–F30) include unimodal functions (F1–F3), simple multimodal functions (F4–F10), hybrid functions (F11–F20), and composition functions (F21–F30) [43,44,45]. The results for functions F1–F10, F11–F20, and F21–F30 are shown in Table 3, Table 4 and Table 5. These tables show the values of different algorithms (EALA, ALA, ACO, GA, DE, FATA, HHO, GJO, GWO, ABC) on the CEC2017 benchmark with 10 dimensions. Each test function contains three statistical indicators: std, avg, and avgtime. The algorithm performance analysis is as follows:

- Average fitness (avg): The average fitness of different algorithms on the same test function varies significantly. Overall, no algorithm has the best average fitness on all test functions. For example, the EALA has a lower average fitness and performs better on some functions, but it has no clear advantage on other functions. This reflects the dependence of algorithm performance on the function; thus, the appropriate algorithm needs to be selected according to the specific function.
- Fitness standard deviation (std): The standard deviation reflects the stability of the algorithm results. For example, on the F6 function, the standard deviation of the ACO algorithm is 5.75 $\times \;10^{-3}$, which is a very small value, indicating that its results on the F6 function are extremely stable; on the F12 function, the standard deviation of the ACO algorithm is 1.45 $\times \;10^{6}$, indicating that its results on this function fluctuate greatly. Some of the algorithms have relatively stable standard deviations on multiple functions, which means that their performance is less affected by function changes. Others have large standard deviation fluctuations, and their stability varies significantly among different functions, so their stability risks need to be considered when using them.
- Average running time (avgtime): The running time of different algorithms varies significantly. On the F1 function, the average running times of the EALA and ACO are 7.10 $\times \;10^{-4}$ and 0.136, respectively. The running time of ACO is much longer than that of the EALA, indicating that the EALA is more efficient in calculating the F1 function. Generally speaking, complex algorithms may take longer to run, but there are exceptions. The running time depends not only on algorithm complexity but also on the function’s characteristics. In practical applications, the solution quality and running time of the algorithm need to be comprehensively considered.

Different algorithms have different advantages and disadvantages in performance (fitness, stability, running time) on different test functions; there is no universal optimal algorithm. In practical applications, it is necessary to comprehensively evaluate and select algorithms based on the requirements of specific problems (corresponding to different test functions), such as the requirements for solution accuracy, stability, and time. If high-precision solutions are sought and time is sufficient, algorithms with good average fitness on the corresponding function can be selected, but it may take a long time; if the process is time-sensitive, algorithms with high computational efficiency and a certain solution quality should be prioritized. These tables provide rich data support for algorithm evaluation and selection.

The Wilcoxon rank-sum test results (Table 6) provide rigorous statistical validation of the EALA’s superior optimization performance on the CEC2017 benchmark. The test reveals statistically significant differences (p<0.05) between the EALA and competing algorithms for 17 of 30 test functions (F3, F4, F11–F15, F17–F19, F23, F26), with particularly strong significance (p≪0.01) observed for complex multimodal and hybrid functions. The results indicate that the performance differences between the EALA and the other algorithms are significant when applied to these functions.

**Figure 7:**
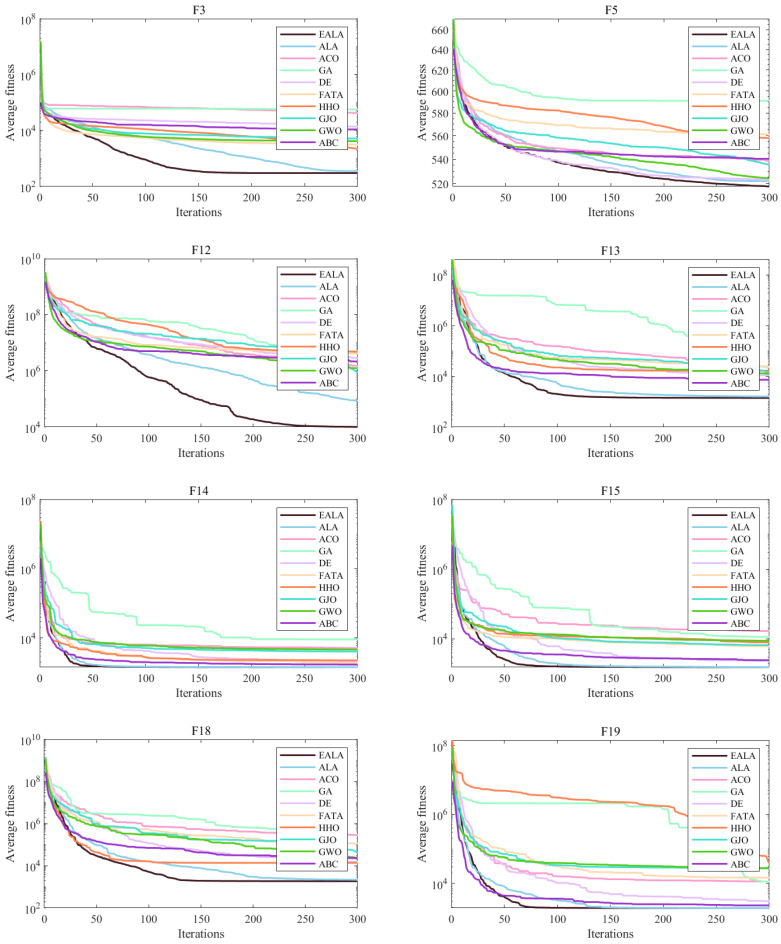
Convergence analysis of partial functions on CECE 2017.

**Figure 8:**
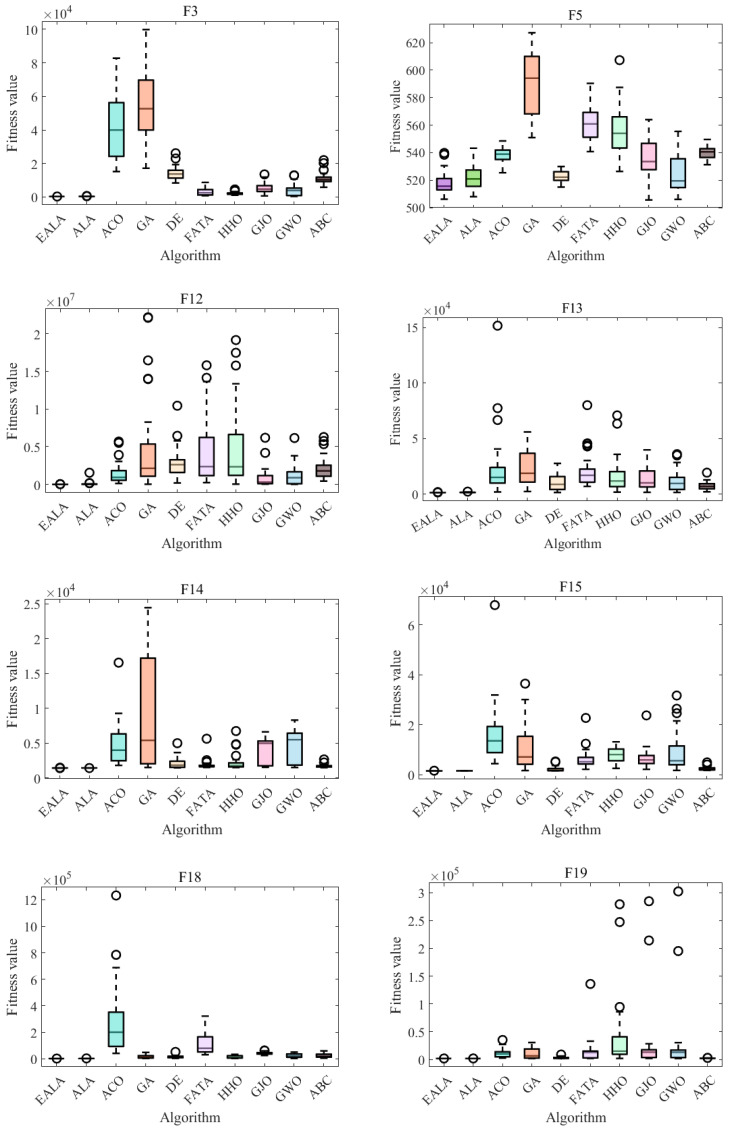
ANOVA test of partial functions on CECE 2017.

In the convergence curve chart (Figure 7), the EALA converges rapidly for most functions. In the boxplot (Figure 8), the fitness values of the EALA are much lower than those of the other algorithms, with a concentrated distribution. For other algorithms, such as DE and HHO, the boxes are at high positions and have many outliers, which is consistent with the poor convergence in the later stage of the line chart. Combining the two types of charts further validates that the EALA significantly outperforms other algorithms in terms of both convergence speed and the stability and optimization results of fitness values across multiple test functions. Other algorithms have problems such as slow convergence, high fitness values, and poor stability (with many outliers) on most test functions.

**Figure 9:**
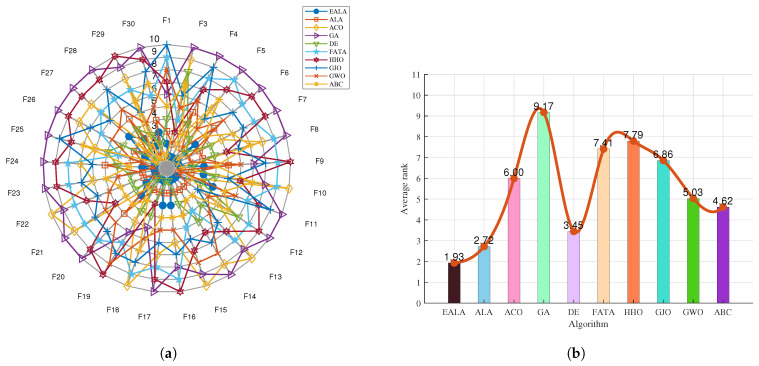
Ranking charts of optimization results on the CEC2017 benchmark. (**a**) The radar chart. (**b**) The average rank chart.

The radar chart (Figure 9a) and ranking analysis (Figure 9b) demonstrate the EALA’s superior and stable performance across the 30 test functions. The EALA achieves the lowest average rank (1.93), significantly outperforming all competitors (e.g., GA at 9.17), while exhibiting a balanced performance distribution (evidenced by evenly spaced radar vertices) and exceptional stability (minimal line fluctuations). The results confirm the EALA’s particular superiority on complex functions (F3, F12–F15, F18–F19, etc.), with its radar vertices extending farthest from the center, highlighting both its optimization capability and robustness compared to alternative approaches.

In conclusion, the enhanced artificial lemming algorithm (EALA) demonstrates remarkable superiority in solving optimization problems. It consistently delivers robust and stable performance across diverse test functions, as evidenced by its top-tier rankings. The data presented in Table 3, Table 4, Table 5 and Table 6, along with the visualizations in Figure 7, Figure 8 and Figure 9, strongly validate the algorithm’s outstanding performance. These results highlight the EALA’s superiority and reliability in handling a broad spectrum of optimization tasks.

Future research will focus on validating the EALA’s effectiveness in real-world engineering applications. Such practical scenarios are characterized by intricate constraints and challenging conditions, which will rigorously test the algorithm’s robustness and practical utility. This extension of the research will help to bridge the gap between theoretical algorithm development and real-world problem solving, further enhancing the EALA’s potential for practical implementation.

## 5. Application of Three-Dimensional Trajectory Planning for Unmanned Aerial Vehicles

This study presents an enhanced multialgorithm fusion framework for the three-dimensional (3D) path planning of UAVs in complex environments. To bridge the gap between simulation and reality, the application component integrated real-world environmental modeling and UAV specifications, including flight dynamics, sensor constraints, and dynamic environmental factors. A high-fidelity 3D terrain model was constructed to formulate the path planning task as a constrained optimization problem. A comprehensive comparative analysis was conducted using eleven optimization algorithms, namely, the EALA, ALA, ACO, GA, DE, FATA, HHO, GJO, PSO, GWO, and ABC, to evaluate their performance in 3D path planning scenarios. The experimental results demonstrate that the proposed approach effectively generates collision-free trajectories while optimizing both the path length and computational efficiency, with the EALA exhibiting superior convergence characteristics and solution quality. The findings contribute novel insights into autonomous UAV navigation in complex 3D operational environments.

### 5.1. Challenges in UAV Path Planning

With the rapid development of UAV technology, its applications in military reconnaissance, disaster rescue, agricultural plant protection, and other fields are becoming increasingly widespread [46]. However, path planning in complex three-dimensional environments remains a key technical challenge restricting autonomous UAV flight [47]. Traditional path planning methods such as the A* algorithm [48] and Dijkstra’s algorithm face problems such as high computational complexity and poor adaptability in 3D environments [49].

In recent years, intelligent optimization algorithms have shown great potential in the field of path planning due to their powerful global search capabilities [50]. In this study, an accurate terrain model was established for complex 3D environments. The path planning problem was transformed into an optimization problem, and the performance of 11 intelligent optimization algorithms was systematically compared. The EALA demonstrated superior performance compared to conventional algorithms in both path quality and computational efficiency, providing new solutions for 3D UAV path planning.

### 5.2. Problem Formulation

#### 5.2.1. Environment Representation

The following equation defines a mathematical model for representing 3D environments in path planning problems:

(15) $$M\in {\left\{0,1\right\}}^{L\times W\times H},\;M\left(i,j,k\right)=\left\{\begin{array}{cc} 1 & \left(\mathrm{Obstacle}\right)\\ 0 & (\mathrm{Free}\;\mathrm{space}) \end{array}\right.$$

The environment is divided into a discrete 3D grid of size L×W×H, where *L*, *W*, and *H* are the number of cells along the length (x-axis), the width (y-axis), and the height (z-axis), respectively. Each cell is indexed by the coordinates (i,j,k), where

i∈[1,L],j∈[1,W],k∈[1,H]

M is a 3D matrix where each element M(i,j,k) is binary: 1 indicates that the cell contains an obstacle (impassable) and 0 means that the cell is free space (traversable).

The proposed algorithm for path planning in 3D environments implements a grid-based search methodology for 3D path planning. It computes a collision-free trajectory from a predefined start point *S* to a target endpoint *E* while considering the following factors:

- Distance cost: the Euclidean distance to the start and goal;
- Priority weights: the terrain safety/threat levels via priority matrix;
- Obstacle constraints: impassable areas marked on a map.

#### 5.2.2. Euclidean Distance Calculation

The actual physical distances between two points in 3D space are used to evaluate the length of the path segment and the closeness between nodes for any two 3D points $p=(p_{x},p_{y},p_{z})$ and $q=(q_{x},q_{y},q_{z})$:

(16) $$D\left(p,q\right)={∥p-q∥}_{2}=\sqrt{{\left(p_{x}-q_{x}\right)}^{2}+{\left(p_{y}-q_{y}\right)}^{2}+{\left(p_{z}-q_{z}\right)}^{2}}$$

### 5.3. The Objective Function of the Algorithm

The path length (L) is ultimately represented by the calculation of the total path length for the total flight distance.

(17) $$\mathrm{Minimize}\;L=\sum_{k=1}^{N-1}{∥p_{k+1}-p_{k}∥}_{2}$$

where $p_{k}$ is the *k*-th point on the path and *N* is the total number of points.

The path curvature variation (S) is expressed by the following formula to represent the smoothness of the path curvature variation and quantify the intensity of changes in the direction of the path (the rate of curvature change). A smaller value indicates a gentler path curvature, which is suitable for high-speed flight.

(18) $$S=\sum_{k=2}^{N-1}{∥\Delta p_{k}-\Delta p_{k-1}∥}_{2}$$

The cumulative threat exposure *R* along a UAV trajectory can be quantified through the following risk assessment formulation:

(19) $$R=\sum_{k=1}^{N}T\left(p_{k}\right)\;\cdot \;\Delta s_{k}$$

where

- $T(p_{k})$ represents the threat intensity at a discrete path point $p_{k}=\left(x_{k},y_{k},z_{k}\right)$, typically normalized to T∈[0,1];
- $\Delta s_{k}={∥p_{k+1}-p_{k}∥}_{2}$ denotes the incremental path length between waypoints.

In the context of path planning for unmanned aerial vehicles (UAVs), the cumulative angular displacement can be quantified as the Turn Count (*T*) defined as the sum of absolute angular changes in both horizontal (azimuthal) and vertical (elevation) planes during trajectory execution. Mathematically, this is expressed as follows:

(20) $$T=\sum_{k=1}^{N-1}\left(|\Delta \psi_{k}|+|\Delta \theta_{k}|\right)$$

where

- $\Delta \psi_{k}=\psi_{k+1}-\psi_{k}$ is the horizontal angle change (azimuth) between waypoints *k* and k+1 (radians);
- $\Delta \theta_{k}=\theta_{k+1}-\theta_{k}$ is the vertical angle change (elevation) between waypoints *k* and k+1 (radians).

The multiobjective cost function (CF) for UAV path planning integrates four critical performance metrics through weighted summation:

(21) $$\mathrm{Cost}=w_{L}L+w_{S}S+w_{R}R+w_{T}T$$

### 5.4. Analysis of Test Results for UAV Path Planning

This paper presents three enhanced artificial lemming algorithms to improve the computational efficiency of sampling-based motion planning algorithms. The proposed methodologies significantly reduce both the search space exploration and planning time while rigorously satisfying all constraints specified in the mathematical formulation.

**Figure 10:**
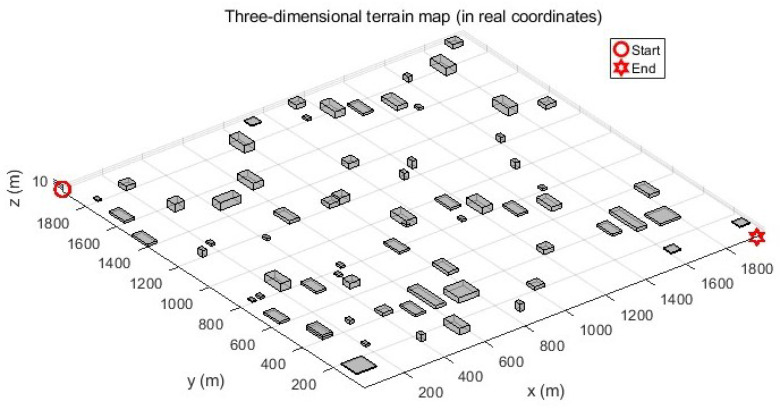
A large-scale three-dimensional terrain environment.

The three-dimensional operational space is discretized using a grid-based mapping approach with two distinct configurations:

- Large-scale environment (Map 1): The experimental environment comprised a volumetric space of 2000m×2000m×40m (length × width × height). Spatial discretization was performed with a resolution of [50,50,1] meters per grid unit along the [x,y,z]-axes, respectively. Obstacles were modeled as parametric cuboids, with geometric parameters (position and dimensions) imported from structured Excel data files. These obstacles were then rendered in the 3D simulation environment. The path planning scenario requires navigation from the initial position at coordinates (1, 1950, 10) to the target location at (1950, 1, 1), as visually represented in Figure 10.
- Medium-scale environment (Map 2): A reduced test environment was configured with the dimensions 1000m×1000m×20m, maintaining the same discretization resolution of [50, 50, 1] meters per grid unit for a consistent comparative analysis. In this configuration, the path planning task initiates at (1, 950, 12) and terminates at (950, 1, 1).

#### 5.4.1. Analysis of Test Results for Large-Scale Environments

**Figure 11:**
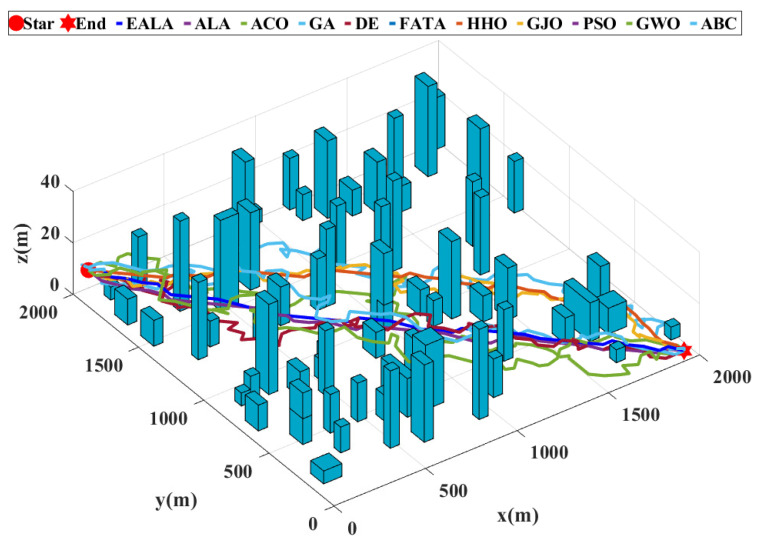
Comparison of UAV path planning for large-scale environments.

This study presents a comprehensive evaluation of UAV path planning algorithms through quantitative metrics and visual analytics with a population size of 10, a maximum iteration number of 20, and specified cost function weights ($w_{L}$: 0.5; $w_{S}$: 0.3; $w_{R}$: 0.1; $w_{T}$: 0.1). The three-dimensional terrain visualization (Figure 11) contextualizes these results, illustrating how the EALA successfully navigates the large-scale complex environment while maintaining optimal path characteristics.

**Figure 12:**
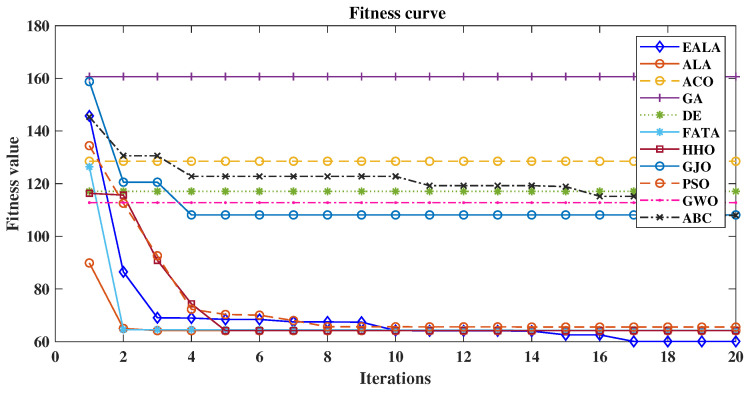
Comparison of fitness curves of UAV path planning for large-scale environments.

As evidenced by the fitness convergence curves (Figure 12), the EALA demonstrates superior optimization characteristics with rapid monotonic convergence, correlating with its best-in-class performance metrics (L = 58.94 (2947 m), S = 25.24 (1262 m), R = 0.00, CT = 1.35 s). The comparative analysis in Table 7 reveals that while PSO shows a competitive path length of 60.26 (3013 m)) and maintains the second-best overall performance, its risk exposure (R = 0.96) and computational stability (evidenced by fitness curve fluctuations) remain suboptimal. Traditional algorithms such as the GA and ABC exhibit fundamental limitations, with the GA producing maximally suboptimal paths (CF = 160.65) and ABC demonstrating pathological computational time (CT = 167.72s), both clearly reflected in their poor convergence behavior. The fitness curves further validate the tabular data, showing that algorithms with nonzero risk (ACO, PSO, etc.) exhibit characteristic instability during optimization, while risk-avoidant algorithms such as the EALA maintain smooth convergence.

These results demonstrate that the EALA’s multiobjective optimization framework successfully balances competing objectives—path length, smoothness, risk avoidance, and computational efficiency—in complex 3D environments. The generated paths are shorter while also exhibiting superior smoothness, reducing energy consumption for UAVs during actual flight. This establishes the EALA as the current state of the art for real-time UAV path planning, particularly in applications requiring strict safety margins and temporal constraints. The systematic comparisons provide quantitative evidence to guide algorithm selection in complex UAV path planning scenarios. Future research should further explore metric trade-offs by incorporating advanced multiobjective optimization theory.

#### 5.4.2. Analysis of Test Results for Medium-Scale Environments

**Figure 13:**
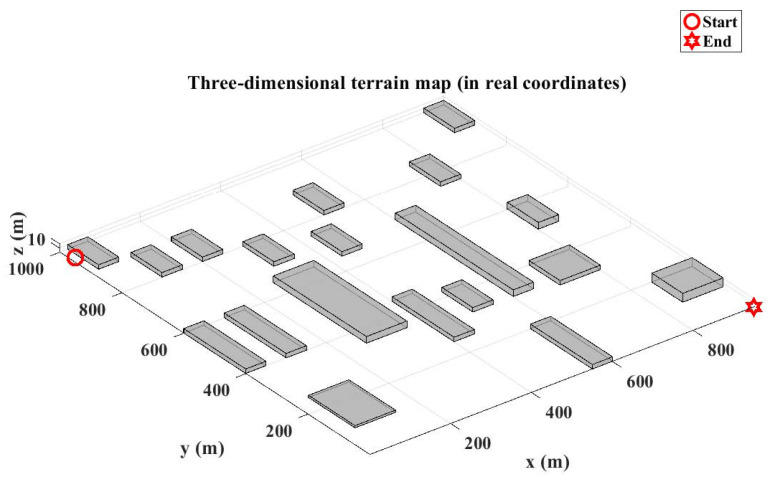
Medium-scale three-dimensional terrain environment.

**Figure 14:**
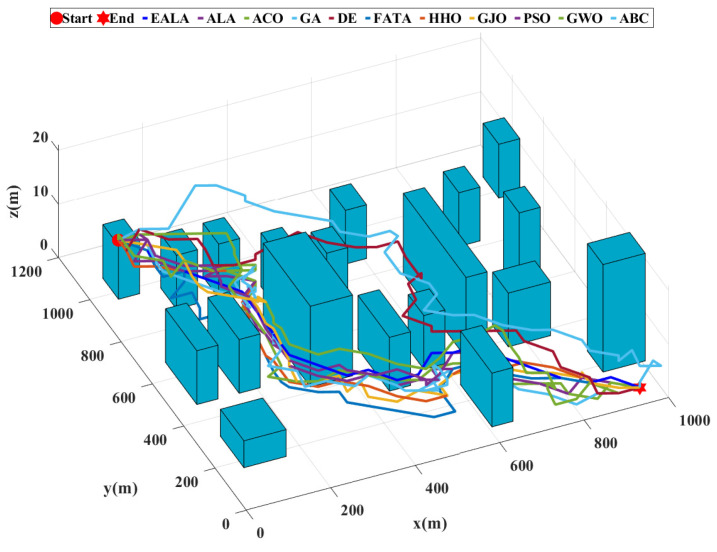
Comparison of UAV path planning for medium-scale environments.

**Figure 15:**
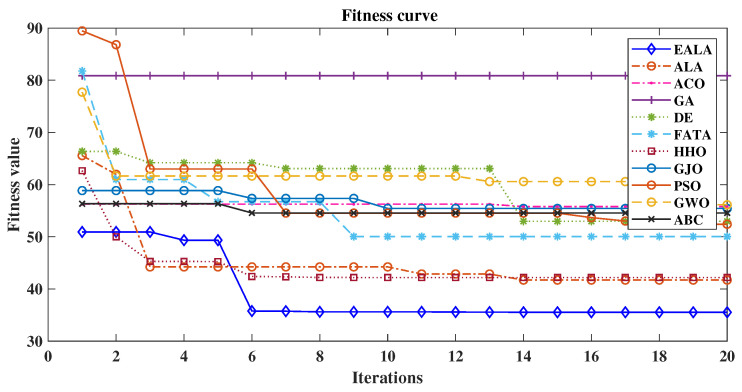
Comparison of fitness curves of UAV path planning for medium-scale environment.

This comprehensive analysis of medium-scale UAV path planning performance, integrating both tabular metrics (Table 8) and visualizations (Figure 13, Figure 14 and Figure 15), demonstrates that the EALA consistently outperforms other algorithms in all critical dimensions. The algorithm maintains its superior performance, with the shortest path length of 34.61m (1730.5m), optimal smoothness of 19.38m (969m), and complete risk avoidance (R=0.00), while achieving real-time computation (CT=10.13s) and a 40% improvement over its closest competitor (HHO at 13.06s).

The fitness curves (Figure 15) reveal the EALA’s characteristically stable exponential convergence, contrasting sharply with the oscillatory behavior of ACO and ABC (CT>150s), while the path visualization (Figure 14) demonstrates its geometrically efficient obstacle negotiation in a medium-scale environment. Notably, while HHO shares the EALA’s perfect risk avoidance, it exhibits 17% longer paths and 29% greater curvature variation.

Traditional algorithms (GA, ACO) show expected limitations, with the GA producing the worst path quality (CF=80.87) despite a moderate runtime and ACO suffering from computational intractability (CT=256.10s). These results validate the EALA as the most robust solution for medium-scale operations, particularly when considering its consistent performance scaling from large-scale environments, though the emergence of the FATA as a potential alternative (CF=50.04) warrants investigation into its adaptive mechanisms.

This study establishes quantitative benchmarks for medium-scale UAV planning while reinforcing the critical relationship between algorithmic architecture and multiobjective performance in constrained 3D spaces.

Herein, a 3D UAV path planning method based on multialgorithm fusion is proposed. The following conclusions were drawn by systematically comparing the performance of 11 optimization algorithms.

- The EALA performs best in terms of path length and convergence speed; it outperforms other algorithms in both large- and medium-scale environments, achieving the shortest, smoothest, and safest paths with real-time computation.
- The EALA proves particularly suitable for time-sensitive operations, with computation times under 2(s) in large-scale scenarios. The algorithm maintains Pareto optimality across all key metrics.

This study establishes the EALA as a state-of-the-art solution for UAV path planning, providing quantitative benchmarks for algorithm selection in both large- and medium-scale 3D environments. The results highlight the critical trade-offs between path quality, safety, and computational efficiency, with the EALA consistently outperforming competing methods.

While the EALA demonstrates superior performance, it exhibits sensitivity to chaotic mapping parameters and faces scalability challenges in large UAV swarms (>50 agents). Its adaptive mechanisms require 3–5 iterations to recover from extreme dynamic disturbances. Nevertheless, the EALA’s core framework shows strong transferability to robotics (trajectory optimization), supply chains (dynamic routing), and disaster response (real-time replanning) through domain-specific constraint reformulation and population-based parallelism, as validated in real-time UAV scenarios (Section 5.4). Future work will focus on automatic parameter tuning and decentralized swarm extensions.

## 6. Conclusions and Prospects

### 6.1. Conclusions

This study introduces the enhanced artificial lemming algorithm (EALA) as a transformative solution to the limitations of traditional optimization algorithms, particularly slow convergence and susceptibility to local optima. The success of the EALA lies in its innovative design philosophy, which integrates chaos theory, adaptive mechanisms, and hybridization strategies. Chaotic initialization based on Kent mapping underscores the significance of starting with a diverse and well-distributed population, which is a fundamental principle applicable to many metaheuristic algorithms aiming to avoid premature convergence. The adaptive random perturbation mechanism and hybrid mutation strategy further demonstrate the effectiveness of dynamic parameter adjustment and algorithmic synergy in tackling complex optimization landscapes.

The benchmark evaluations on the IEEE CEC2017 and CEC2022 test suites yield a critical insight: algorithms that can adaptively balance global exploration and local exploitation across varying problem complexities tend to outperform their static counterparts. The EALA’s superior performance metrics—manifested in high solution quality, stability, and computational efficiency—highlight the generalizable value of combining multiple optimization techniques. The Wilcoxon rank-sum tests and ablation studies not only validate the EALA’s statistical superiority but also offer empirical evidence for the modular design approach, where each enhancement contributes both individually and synergistically.

In the context of 3D UAV path planning, the EALA’s success showcases the practical relevance of metaheuristic algorithms in real-world engineering challenges. It demonstrates that algorithms capable of optimizing multiple conflicting objectives (e.g., path length, smoothness, and threat exposure) while meeting real-time constraints can bridge the gap between theoretical research and practical applications. This finding suggests that similar hybrid optimization frameworks could be effective in other complex engineering domains characterized by multiple constraints and dynamic environments.

### 6.2. Prospects

Looking ahead, the EALA and related optimization algorithms should be developed in three key areas. First, advancing the integration of chaos theory requires exploring more sophisticated chaotic mapping models that can adapt to different problem structures, potentially through the development of self-adaptive chaotic sequence generators. Second, parameter tuning mechanisms should evolve toward greater autonomy, leveraging machine learning techniques to automatically adjust algorithmic parameters based on a real-time analysis of the search process. This would enable optimization algorithms to dynamically respond to problem complexity without manual intervention.

From an application perspective, future research should aim to transfer the EALA’s success in UAV path planning to other critical sectors. This involves developing domain-specific adaptations that can handle the unique constraints of intelligent transportation, energy systems, and mechanical design. Moreover, the combination of multiobjective optimization with emerging machine learning paradigms, such as deep reinforcement learning, presents an exciting opportunity to create intelligent hybrid frameworks capable of continuous learning and adaptation. Finally, reducing computational complexity remains a paramount challenge. Future efforts should explore approximation techniques, parallel computing architectures, and model-based optimization methods to make advanced algorithms such as the EALA more scalable for large-scale problems. By addressing these aspects, optimization algorithms can become even more powerful tools for solving the complex challenges of the digital age.

## Figures and Tables

**Table 1:** Std, avg, and avgtime for 10 dimensions in the CEC2022 benchmark.

Func.	Type	EALA	ALA	ACO	GA	DE	FATA	HHO	GJO	GWO	ABC
F1	std	4.47 × 10^3^	5.11 × 10^3^	1.49 × 10^5^	2.13 × 10^4^	1.01 × 10^4^	7.55 × 10^3^	1.34 × 10^4^	5.94 × 10^3^	7.97 × 10^3^	7.48 × 10^3^
	avg	7.25 × 10^3^	1.09 × 10^4^	3.15 × 10^5^	8.60 × 10^4^	5.27 × 10^4^	1.78 × 10^4^	3.62 × 10^4^	1.67 × 10^4^	1.92 × 10^4^	4.62 × 10^4^
	avgtime	1.04 × 10^−3^	9.29 × 10^−4^	2.81 × 10^−1^	5.21 × 10^−2^	1.08 × 10^−1^	3.53 × 10^−2^	5.05 × 10^−2^	5.36 × 10^−2^	4.15 × 10^−2^	1.41 × 10^−1^
F2	std	2.42 × 10^1^	2.47 × 10^1^	1.39	1.87 × 10^2^	1.28 × 10^1^	7.29 × 10^1^	6.16 × 10^1^	7.80 × 10^1^	3.59 × 10^1^	9.14
	avg	4.66 × 10^2^	4.63 × 10^2^	4.20 × 10^2^	8.06 × 10^2^	4.61 × 10^2^	6.50 × 10^2^	5.92 × 10^2^	6.21 × 10^2^	5.07 × 10^2^	4.62 × 10^2^
	avgtime	9.07 × 10^−4^	8.19 × 10^−4^	2.57 × 10^−1^	5.37 × 10^−2^	1.12 × 10^−1^	3.40 × 10^−2^	4.71 × 10^−2^	5.09 × 10^−2^	4.12 × 10^−2^	1.48 × 10^−1^
F3	std	3.20	7.72	1.55	1.20 × 10^1^	2.27 × 10^−1^	8.10	1.03 × 10^1^	1.33 × 10^1^	6.15	1.03
	avg	6.06 × 10^2^	6.09 × 10^2^	6.04 × 10^2^	7.01 × 10^2^	6.01 × 10^2^	6.67 × 10^2^	6.64 × 10^2^	6.30 × 10^2^	6.09 × 10^2^	6.04 × 10^2^
	avgtime	2.15 × 10^−3^	1.87 × 10^−3^	3.11 × 10^−1^	8.28 × 10^−2^	1.48 × 10^−1^	6.06 × 10^−2^	1.22 × 10^−1^	7.67 × 10^−2^	6.95 × 10^−2^	2.08 × 10^−1^
F4	std	2.25 × 10^1^	2.28 × 10^1^	1.09 × 10^1^	2.29 × 10^1^	9.45	1.42 × 10^1^	1.58 × 10^1^	3.38 × 10^1^	2.78 × 10^1^	1.02 × 10^1^
	avg	8.72 × 10^2^	8.72 × 10^2^	9.47 × 10^2^	1.02 × 10^3^	9.42 × 10^2^	9.50 × 10^2^	8.85 × 10^2^	9.15 × 10^2^	8.63 × 10^2^	9.38 × 10^2^
	avgtime	1.34 × 10^−3^	1.30 × 10^−3^	3.11 × 10^−1^	7.15 × 10^−2^	1.16 × 10^−1^	3.93 × 10^−2^	7.18 × 10^−2^	5.90 × 10^−2^	4.80 × 10^−2^	1.61 × 10^−1^
F5	std	3.39 × 10^2^	3.89 × 10^2^	1.21 × 10^2^	1.21 × 10^3^	4.74 × 10^2^	4.12 × 10^2^	3.74 × 10^2^	5.69 × 10^2^	4.01 × 10^2^	1.66 × 10^2^
	avg	1.21 × 10^3^	1.40 × 10^3^	1.13 × 10^3^	2.68 × 10^3^	2.34 × 10^3^	3.25 × 10^3^	2.98 × 10^3^	2.38 × 10^3^	1.40 × 10^3^	1.36 × 10^3^
	avgtime	1.43 × 10^−3^	1.47 × 10^−3^	2.84 × 10^−1^	6.47 × 10^−2^	1.18 × 10^−1^	4.34 × 10^−2^	7.42 × 10^−2^	6.00 × 10^−2^	5.01 × 10^−2^	1.65 × 10^−1^
F6	std	7.49 × 10^3^	1.00 × 10^4^	4.75 × 10^7^	1.09 × 10^6^	1.06 × 10^7^	8.72 × 10^6^	2.41 × 10^5^	7.79 × 10^7^	6.76 × 10^6^	5.44 × 10^6^
	avg	8.01 × 10^3^	1.70 × 10^4^	9.19 × 10^7^	7.71 × 10^5^	1.81 × 10^7^	1.60 × 10^7^	3.96 × 10^5^	3.24 × 10^7^	2.79 × 10^6^	1.17 × 10^7^
	avgtime	1.29 × 10^−3^	9.19 × 10^−4^	2.43 × 10^−1^	5.70 × 10^−2^	1.28 × 10^−1^	3.72 × 10^−2^	5.55 × 10^−2^	5.50 × 10^−2^	4.36 × 10^−2^	1.52 × 10^−1^
F7	std	3.77 × 10^1^	4.08 × 10^1^	2.91 × 10^1^	7.15 × 10^1^	1.54 × 10^1^	3.30 × 10^1^	5.33 × 10^1^	3.98 × 10^1^	6.13 × 10^1^	2.15 × 10^1^
	avg	2.10 × 10^3^	2.09 × 10^3^	2.20 × 10^3^	2.24 × 10^3^	2.08 × 10^3^	2.19 × 10^3^	2.20 × 10^3^	2.12 × 10^3^	2.12 × 10^3^	2.14 × 10^3^
	avgtime	2.44 × 10^−3^	2.03 × 10^−3^	3.11 × 10^−1^	9.27 × 10^−2^	1.49 × 10^−1^	6.47 × 10^−2^	1.38 × 10^−1^	8.60 × 10^−2^	7.55 × 10^−2^	2.27 × 10^−1^
F8	std	1.82 × 10^1^	3.61 × 10^1^	5.23 × 10^1^	1.09 × 10^2^	3.36	3.72 × 10^1^	9.88 × 10^1^	6.50 × 10^1^	6.84 × 10^1^	7.67
	avg	2.23 × 10^3^	2.24 × 10^3^	2.38 × 10^3^	2.38 × 10^3^	2.23 × 10^3^	2.27 × 10^3^	2.30 × 10^3^	2.28 × 10^3^	2.28 × 10^3^	2.25 × 10^3^
	avgtime	2.51 × 10^−3^	2.57 × 10^−3^	3.41 × 10^−1^	1.05 × 10^−1^	1.61 × 10^−1^	7.79 × 10^−2^	1.58 × 10^−1^	9.09 × 10^−2^	8.56 × 10^−2^	2.49 × 10^−1^
F9	std	5.25 × 10^−1^	2.79	5.86 × 10^1^	8.19 × 10^1^	2.26 × 10^−1^	3.53 × 10^1^	5.52 × 10^1^	3.92 × 10^1^	3.94 × 10^1^	7.46 × 10^−1^
	avg	2.48 × 10^3^	2.48 × 10^3^	2.55 × 10^3^	2.75 × 10^3^	2.48 × 10^3^	2.61 × 10^3^	2.59 × 10^3^	2.58 × 10^3^	2.53 × 10^3^	2.48 × 10^3^
	avgtime	2.35 × 10^−3^	2.46 × 10^−3^	3.21 × 10^−1^	1.01 × 10^−1^	2.06 × 10^−1^	6.60 × 10^−2^	1.30 × 10^−1^	9.23 × 10^−2^	6.81 × 10^−2^	2.40 × 10^−1^
F10	std	1.27 × 10^3^	9.77 × 10^2^	2.86 × 10^2^	1.28 × 10^3^	4.50 × 10^2^	1.73 × 10^3^	7.54 × 10^2^	1.79 × 10^3^	6.81 × 10^2^	5.83 × 10^2^
	avg	4.32 × 10^3^	3.88 × 10^3^	7.40 × 10^3^	6.02 × 10^3^	2.84 × 10^3^	5.03 × 10^3^	4.53 × 10^3^	4.36 × 10^3^	3.59 × 10^3^	2.69 × 10^3^
	avgtime	2.12 × 10^−3^	2.16 × 10^−3^	3.32 × 10^−1^	9.19 × 10^−2^	1.69 × 10^−1^	6.41 × 10^−2^	1.30 × 10^−1^	8.46 × 10^−2^	7.00 × 10^−2^	2.08 × 10^−1^
F11	std	4.23 × 10^1^	1.34 × 10^2^	1.26 × 10^2^	1.49 × 10^4^	2.24 × 10^1^	7.08 × 10^2^	7.68 × 10^2^	8.28 × 10^2^	4.45 × 10^2^	1.69 × 10^2^
	avg	3.03 × 10^3^	3.21 × 10^3^	3.35 × 10^3^	2.70 × 10^4^	3.00 × 10^3^	6.60 × 10^3^	4.42 × 10^3^	5.84 × 10^3^	3.80 × 10^3^	3.64 × 10^3^
	avgtime	2.94 × 10^−3^	2.54 × 10^−3^	3.35 × 10^−1^	1.14 × 10^−1^	1.79 × 10^−1^	8.71 × 10^−2^	1.63 × 10^−1^	9.86 × 10^−2^	9.27 × 10^−2^	2.58 × 10^−1^
F12	std	3.02 × 10^1^	2.91 × 10^1^	3.41 × 10^−5^	1.82 × 10^2^	4.54	1.74 × 10^2^	1.66 × 10^2^	6.55 × 10^1^	2.92 × 10^1^	4.91
	avg	2.97 × 10^3^	2.96 × 10^3^	2.90 × 10^3^	3.62 × 10^3^	2.95 × 10^3^	3.29 × 10^3^	3.28 × 10^3^	3.05 × 10^3^	2.98 × 10^3^	2.96 × 10^3^
	avgtime	2.97 × 10^−3^	3.31 × 10^−3^	3.40 × 10^−1^	1.17 × 10^−1^	1.77 × 10^−1^	8.81 × 10^−2^	1.90 × 10^−1^	1.06 × 10^−1^	9.84 × 10^−2^	2.60 × 10^−1^

**Table 2:** The results of the Wilcoxon rank-sum test on the CEC2022 benchmark with 10 dimensions.

Func.	ALA	ACO	GA	DE	FATA	HHO	GJO	GWO	ABC
F1	3.85 × 10^−3^	3.02 × 10^−11^	3.02 × 10^−11^	3.02 × 10^−11^	1.20 × 10^−8^	3.34 × 10^−11^	4.69 × 10^−8^	2.39 × 10^−8^	3.02 × 10^−11^
F2	8.77 × 10^−1^	3.02 × 10^−11^	3.34 × 10^−11^	6.84 × 10^−1^	3.69 × 10^−11^	1.21 × 10^−10^	2.61 × 10^−10^	4.44 × 10^−7^	7.28 × 10^−1^
F3	7.73 × 10^−2^	1.24 × 10^−3^	3.02 × 10^−11^	3.02 × 10^−11^	3.02 × 10^−11^	3.02 × 10^−11^	1.09 × 10^−10^	7.01 × 10^−2^	1.99 × 10^−2^
F4	9.59 × 10^−1^	3.02 × 10^−11^	3.02 × 10^−11^	3.02 × 10^−11^	3.02 × 10^−11^	2.42 × 10^−2^	6.74 × 10^−6^	4.36 × 10^−2^	3.02 × 10^−11^
F5	2.32 × 10^−2^	9.47 × 10^−1^	1.86 × 10^−9^	3.16 × 10^−10^	3.69 × 10^−11^	4.08 × 10^−11^	6.12 × 10^−10^	1.03 × 10^−2^	1.49 × 10^−4^
F6	5.97 × 10^−5^	3.02 × 10^−11^	3.02 × 10^−11^	3.02 × 10^−11^	3.02 × 10^−11^	3.02 × 10^−11^	3.02 × 10^−11^	1.96 × 10^−10^	3.02 × 10^−11^
F7	3.71 × 10^−1^	6.12 × 10^−10^	1.78 × 10^−10^	2.06 × 10^−1^	8.10 × 10^−10^	5.46 × 10^−9^	5.08 × 10^−3^	2.64 × 10^−1^	1.87 × 10^−5^
F8	4.29 × 10^−1^	4.50 × 10^−11^	5.07 × 10^−10^	8.53 × 10^−1^	1.85 × 10^−8^	9.51 × 10^−6^	7.62 × 10^−3^	2.40 × 10^−1^	1.56 × 10^−8^
F9	3.78 × 10^−2^	3.02 × 10^−11^	3.02 × 10^−11^	1.49 × 10^−6^	3.02 × 10^−11^	3.02 × 10^−11^	3.02 × 10^−11^	3.69 × 10^−11^	1.09 × 10^−10^
F10	7.01 × 10^−2^	3.02 × 10^−11^	1.47 × 10^−7^	3.18 × 10^−3^	5.19 × 10^−2^	9.94 × 10^−1^	2.71 × 10^−1^	4.23 × 10^−3^	5.32 × 10^−3^
F11	9.26 × 10^−9^	3.02 × 10^−11^	3.02 × 10^−11^	2.81 × 10^−2^	3.02 × 10^−11^	3.02 × 10^−11^	3.02 × 10^−11^	3.02 × 10^−11^	3.02 × 10^−11^
F12	7.17 × 10^−1^	3.02 × 10^−11^	3.02 × 10^−11^	8.77 × 10^−2^	3.02 × 10^−11^	7.39 × 10^−11^	8.35 × 10^−8^	2.07 × 10^−2^	8.30 × 10^−1^

**Table 3:** Std, avg, and avgtime for 10 dimensions on the CEC2017 benchmark (F1–F10).

Func.	Type	EALA	ALA	ACO	GA	DE	FATA	HHO	GJO	GWO	ABC
F1	std	3.64 × 10^3^	4.12 × 10^3^	2.80 × 10^3^	6.96 × 10^6^	8.06 × 10^4^	1.37 × 10^8^	2.47 × 10^7^	4.21 × 10^8^	8.85 × 10^7^	2.55 × 10^6^
	avg	4.39 × 10^3^	5.07 × 10^3^	2.08 × 10^3^	5.31 × 10^6^	8.96 × 10^4^	2.64 × 10^8^	1.66 × 10^7^	5.05 × 10^8^	2.59 × 10^7^	1.27 × 10^6^
	avgtime	7.10 × 10^−4^	6.37 × 10^−4^	1.36 × 10^−1^	5.11 × 10^−2^	9.79 × 10^−2^	2.17 × 10^−2^	3.17 × 10^−2^	3.11 × 10^−2^	2.10 × 10^−2^	1.15 × 10^−1^
F3	std	4.44 × 10^−2^	7.95 × 10^1^	2.00 × 10^4^	2.22 × 10^4^	4.20 × 10^3^	2.19 × 10^3^	9.40 × 10^2^	3.14 × 10^3^	3.63 × 10^3^	3.50 × 10^3^
	avg	3.00 × 10^2^	3.54 × 10^2^	4.23 × 10^4^	5.67 × 10^4^	1.38 × 10^4^	3.15 × 10^3^	2.22 × 10^3^	5.17 × 10^3^	4.21 × 10^3^	1.09 × 10^4^
	avgtime	6.91 × 10^−4^	5.84 × 10^−4^	1.27 × 10^−1^	3.96 × 10^−2^	8.07 × 10^−2^	2.10 × 10^−2^	3.08 × 10^−2^	3.08 × 10^−2^	2.04 × 10^−2^	1.10 × 10^−1^
F4	std	1.19 × 10^1^	1.21 × 10^1^	1.66 × 10^−1^	7.02 × 10^1^	8.28 × 10^−1^	2.77 × 10^1^	4.05 × 10^1^	4.46 × 10^1^	1.84 × 10^1^	4.34 × 10^−1^
	avg	4.05 × 10^2^	4.07 × 10^2^	4.06 × 10^2^	4.95 × 10^2^	4.07 × 10^2^	4.45 × 10^2^	4.44 × 10^2^	4.51 × 10^2^	4.19 × 10^2^	4.07 × 10^2^
	avgtime	6.94 × 10^−4^	5.95 × 10^−4^	1.25 × 10^−1^	4.03 × 10^−2^	8.15 × 10^−2^	2.08 × 10^−2^	3.13 × 10^−2^	3.07 × 10^−2^	2.04 × 10^−2^	1.15 × 10^−1^
F5	std	7.91	8.12	5.04	2.24 × 10^1^	4.06	1.21 × 10^1^	1.89 × 10^1^	1.39 × 10^1^	1.29 × 10^1^	4.53
	avg	5.17 × 10^2^	5.21 × 10^2^	5.38 × 10^2^	5.91 × 10^2^	5.22 × 10^2^	5.60 × 10^2^	5.57 × 10^2^	5.35 × 10^2^	5.24 × 10^2^	5.40 × 10^2^
	avgtime	7.91 × 10^−4^	7.16 × 10^−4^	1.29 × 10^−1^	4.33 × 10^−2^	8.46 × 10^−2^	2.30 × 10^−2^	4.17 × 10^−2^	3.39 × 10^−2^	2.37 × 10^−2^	1.18 × 10^−1^
F6	std	1.38 × 10^−1^	4.34 × 10^−1^	5.75 × 10^−5^	9.36	3.27 × 10^−3^	1.12 × 10^1^	1.15 × 10^1^	8.53	2.41	1.48 × 10^−1^
	avg	6.00 × 10^2^	6.00 × 10^2^	6.00 × 10^2^	6.64 × 10^2^	6.00 × 10^2^	6.34 × 10^2^	6.44 × 10^2^	6.11 × 10^2^	6.01 × 10^2^	6.00 × 10^2^
	avgtime	1.13 × 10^−3^	1.03 × 10^−3^	1.40 × 10^−1^	5.48 × 10^−2^	9.71 × 10^−2^	3.17 × 10^−2^	6.10 × 10^−2^	4.23 × 10^−2^	3.25 × 10^−2^	1.38 × 10^−1^
F7	std	7.29	8.60	5.46	3.69 × 10^1^	3.45	1.79 × 10^1^	2.24 × 10^1^	1.46 × 10^1^	9.65	5.90
	avg	7.29 × 10^2^	7.35 × 10^2^	7.50 × 10^2^	8.42 × 10^2^	7.36 × 10^2^	7.82 × 10^2^	7.93 × 10^2^	7.59 × 10^2^	7.32 × 10^2^	7.51 × 10^2^
	avgtime	8.53 × 10^−4^	7.34 × 10^−4^	1.31 × 10^−1^	4.49 × 10^−2^	8.60 × 10^−2^	2.43 × 10^−2^	4.39 × 10^−2^	3.48 × 10^−2^	2.42 × 10^−2^	1.21 × 10^−1^
F8	std	9.04	8.97	6.28	1.70 × 10^1^	3.92	6.19	1.11 × 10^1^	1.28 × 10^1^	7.90	4.98
	avg	8.18 × 10^2^	8.21 × 10^2^	8.38 × 10^2^	8.81 × 10^2^	8.25 × 10^2^	8.40 × 10^2^	8.32 × 10^2^	8.35 × 10^2^	8.18 × 10^2^	8.38 × 10^2^
	avgtime	8.20 × 10^−4^	7.06 × 10^−4^	1.32 × 10^−1^	4.39 × 10^−2^	8.64 × 10^−2^	2.33 × 10^−2^	4.26 × 10^−2^	3.39 × 10^−2^	2.42 × 10^−2^	1.18 × 10^−1^
F9	std	1.41	3.12	9.06 × 10^−7^	7.37 × 10^1^	3.38 × 10^−1^	1.12 × 10^2^	2.18 × 10^2^	5.92 × 10^1^	2.55 × 10^1^	2.08
	avg	9.00 × 10^2^	9.02 × 10^2^	9.00 × 10^2^	9.84 × 10^2^	9.00 × 10^2^	1.05 × 10^3^	1.52 × 10^3^	1.00 × 10^3^	9.22 × 10^2^	9.02 × 10^2^
	avgtime	8.34 × 10^−4^	7.25 × 10^−4^	1.33 × 10^−1^	4.42 × 10^−2^	8.77 × 10^−2^	2.44 × 10^−2^	4.42 × 10^−2^	3.45 × 10^−2^	2.44 × 10^−2^	1.20 × 10^−1^
F10	std	2.63 × 10^2^	2.52 × 10^2^	1.69 × 10^2^	3.37 × 10^2^	1.52 × 10^2^	2.05 × 10^2^	2.67 × 10^2^	3.89 × 10^2^	3.26 × 10^2^	1.75 × 10^2^
	avg	1.88 × 10^3^	1.79 × 10^3^	2.91 × 10^3^	2.50 × 10^3^	2.00 × 10^3^	2.70 × 10^3^	2.07 × 10^3^	2.07 × 10^3^	1.73 × 10^3^	2.40 × 10^3^
	avgtime	8.98 × 10^−4^	7.94 × 10^−4^	1.35 × 10^−1^	4.83 × 10^−2^	9.14 × 10^−2^	2.48 × 10^−2^	4.82 × 10^−2^	3.60 × 10^−2^	2.55 × 10^−2^	1.23 × 10^−1^

**Table 4:** Std, avg, and avgtime for 10 dimensions on the CEC2022 benchmark (F11–F20).

Func.	Type	EALA	ALA	ACO	GA	DE	FATA	HHO	GJO	GWO	ABC
F11	std	3.97 × 10^1^	5.38	3.00	6.77 × 10^3^	3.70	4.75 × 10^1^	8.65 × 10^1^	7.93 × 10^2^	4.65 × 10^1^	3.66
	avg	1.11 × 10^3^	1.11 × 10^3^	1.11 × 10^3^	6.37 × 10^3^	1.11 × 10^3^	1.19 × 10^3^	1.20 × 10^3^	1.33 × 10^3^	1.15 × 10^3^	1.11 × 10^3^
	avgtime	7.79 × 10^−4^	6.55 × 10^−4^	1.29 × 10^−1^	4.14 × 10^−2^	8.41 × 10^−2^	2.24 × 10^−2^	3.66 × 10^−2^	3.20 × 10^−2^	2.21 × 10^−2^	1.16 × 10^−1^
F12	std	1.40 × 10^4^	2.80 × 10^5^	1.45 × 10^6^	6.32 × 10^6^	2.07 × 10^6^	4.32 × 10^6^	5.40 × 10^6^	1.32 × 10^6^	1.43 × 10^6^	1.48 × 10^6^
	avg	1.00 × 10^4^	8.82 × 10^4^	1.51 × 10^6^	5.00 × 10^6^	2.82 × 10^6^	4.15 × 10^6^	4.81 × 10^6^	8.87 × 10^5^	1.23 × 10^6^	2.14 × 10^6^
	avgtime	8.07 × 10^−4^	6.52 × 10^−4^	1.30 × 10^−1^	4.68 × 10^−2^	9.14 × 10^−2^	2.34 × 10^−2^	3.99 × 10^−2^	3.43 × 10^−2^	2.31 × 10^−2^	1.38 × 10^−1^
F13	std	1.24 × 10^2^	2.60 × 10^2^	2.96 × 10^4^	1.47 × 10^4^	7.60 × 10^3^	1.56 × 10^4^	1.65 × 10^4^	1.06 × 10^4^	1.02 × 10^4^	3.76 × 10^3^
	avg	1.41 × 10^3^	1.61 × 10^3^	2.43 × 10^4^	2.30 × 10^4^	1.08 × 10^4^	2.17 × 10^4^	1.68 × 10^4^	1.39 × 10^4^	1.27 × 10^4^	7.42 × 10^3^
	avgtime	9.54 × 10^−4^	1.23 × 10^−3^	1.69 × 10^−1^	5.11 × 10^−2^	1.05 × 10^−1^	2.76 × 10^−2^	5.03 × 10^−2^	3.94 × 10^−2^	2.90 × 10^−2^	1.47 × 10^−1^
F14	std	1.54 × 10^1^	4.94	3.24 × 10^3^	7.95 × 10^3^	7.57 × 10^2^	7.41 × 10^2^	1.18 × 10^3^	1.81 × 10^3^	2.25 × 10^3^	2.36 × 10^2^
	avg	1.42 × 10^3^	1.43 × 10^3^	5.05 × 10^3^	9.08 × 10^3^	2.05 × 10^3^	1.89 × 10^3^	2.24 × 10^3^	4.02 × 10^3^	4.63 × 10^3^	1.70 × 10^3^
	avgtime	1.04 × 10^−3^	8.86 × 10^−4^	1.72 × 10^−1^	5.73 × 10^−2^	1.14 × 10^−1^	3.25 × 10^−2^	5.81 × 10^−2^	4.38 × 10^−2^	3.09 × 10^−2^	1.56 × 10^−1^
F15	std	3.05 × 10^1^	2.37 × 10^1^	1.26 × 10^4^	1.00 × 10^4^	1.10 × 10^3^	3.83 × 10^3^	3.09 × 10^3^	3.98 × 10^3^	7.91 × 10^3^	7.06 × 10^2^
	avg	1.53 × 10^3^	1.54 × 10^3^	1.67 × 10^4^	1.13 × 10^4^	2.36 × 10^3^	6.08 × 10^3^	7.89 × 10^3^	6.57 × 10^3^	8.84 × 10^3^	2.44 × 10^3^
	avgtime	8.90 × 10^−4^	7.48 × 10^−4^	1.49 × 10^−1^	4.09 × 10^−2^	8.55 × 10^−2^	2.67 × 10^−2^	4.72 × 10^−2^	4.12 × 10^−2^	2.69 × 10^−2^	1.45 × 10^−1^
F15	std	3.05 × 10^1^	2.37 × 10^1^	1.26 × 10^4^	1.00 × 10^4^	1.10 × 10^3^	3.83 × 10^3^	3.09 × 10^3^	3.98 × 10^3^	7.91 × 10^3^	7.06 × 10^2^
	avg	1.53 × 10^3^	1.54 × 10^3^	1.67 × 10^4^	1.13 × 10^4^	2.36 × 10^3^	6.08 × 10^3^	7.89 × 10^3^	6.57 × 10^3^	8.84 × 10^3^	2.44 × 10^3^
	avgtime	8.90 × 10^−4^	7.48 × 10^−4^	1.49 × 10^−1^	4.09 × 10^−2^	8.55 × 10^−2^	2.67 × 10^−2^	4.72 × 10^−2^	4.12 × 10^−2^	2.69 × 10^−2^	1.45 × 10^−1^
F16	std	5.69 × 10^1^	5.31 × 10^1^	5.73 × 10^1^	1.24 × 10^2^	3.83 × 10^1^	1.49 × 10^2^	1.25 × 10^2^	1.44 × 10^2^	1.10 × 10^2^	2.88 × 10^1^
	avg	1.66 × 10^3^	1.66 × 10^3^	1.75 × 10^3^	1.88 × 10^3^	1.65 × 10^3^	1.90 × 10^3^	1.97 × 10^3^	1.86 × 10^3^	1.73 × 10^3^	1.67 × 10^3^
	avgtime	9.90 × 10^−4^	9.19 × 10^−4^	1.64 × 10^−1^	5.49 × 10^−2^	1.03 × 10^−1^	2.94 × 10^−2^	5.06 × 10^−2^	4.28 × 10^−2^	2.83 × 10^−2^	1.54 × 10^−1^
F17	std	1.57 × 10^1^	1.22 × 10^1^	2.83 × 10^1^	6.93 × 10^1^	8.49	2.49 × 10^1^	7.60 × 10^1^	3.54 × 10^1^	2.72 × 10^1^	1.27 × 10^1^
	avg	1.75 × 10^3^	1.74 × 10^3^	1.78 × 10^3^	1.80 × 10^3^	1.73 × 10^3^	1.80 × 10^3^	1.80 × 10^3^	1.77 × 10^3^	1.77 × 10^3^	1.77 × 10^3^
	avgtime	1.22 × 10^−3^	1.30 × 10^−3^	1.77 × 10^−1^	6.08 × 10^−2^	1.09 × 10^−1^	3.84 × 10^−2^	7.17 × 10^−2^	4.86 × 10^−2^	3.54 × 10^−2^	1.65 × 10^−1^
F18	std	4.96 × 10^1^	2.87 × 10^2^	2.81 × 10^5^	1.20 × 10^4^	9.59 × 10^3^	7.91 × 10^4^	1.02 × 10^4^	1.07 × 10^4^	1.33 × 10^4^	1.41 × 10^4^
	avg	1.86 × 10^3^	2.18 × 10^3^	2.89 × 10^5^	1.59 × 10^4^	1.47 × 10^4^	1.11 × 10^5^	1.39 × 10^4^	4.20 × 10^4^	2.31 × 10^4^	2.31 × 10^4^
	avgtime	1.01 × 10^−3^	8.96 × 10^−4^	1.65 × 10^−1^	5.53 × 10^−2^	1.01 × 10^−1^	2.93 × 10^−2^	5.02 × 10^−2^	4.14 × 10^−2^	3.13 × 10^−2^	1.49 × 10^−1^
F19	std	7.98	1.69 × 10^1^	7.94 × 10^3^	9.94 × 10^3^	1.54 × 10^3^	2.42 × 10^4^	6.52 × 10^4^	6.15 × 10^4^	6.21 × 10^4^	2.84 × 10^2^
	avg	1.90 × 10^3^	1.91 × 10^3^	1.12 × 10^4^	1.12 × 10^4^	3.01 × 10^3^	1.46 × 10^4^	4.08 × 10^4^	2.70 × 10^4^	2.76 × 10^4^	2.27 × 10^3^
	avgtime	2.98 × 10^−3^	2.21 × 10^−3^	2.04 × 10^−1^	1.10 × 10^−1^	1.59 × 10^−1^	8.69 × 10^−2^	1.91 × 10^−1^	1.01 × 10^−1^	8.52 × 10^−2^	2.68 × 10^−1^
F20	std	3.35 × 10^1^	4.93 × 10^1^	2.53 × 10^1^	8.32 × 10^1^	4.66	5.38 × 10^1^	7.41 × 10^1^	7.01 × 10^1^	7.02 × 10^1^	1.06 × 10^1^
	avg	2.04 × 10^3^	2.05 × 10^3^	2.04 × 10^3^	2.25 × 10^3^	2.00 × 10^3^	2.18 × 10^3^	2.19 × 10^3^	2.13 × 10^3^	2.10 × 10^3^	2.05 × 10^3^
	avgtime	1.27 × 10^−3^	1.04 × 10^−3^	1.75 × 10^−1^	6.28 × 10^−2^	1.17 × 10^−1^	3.62 × 10^−2^	7.15 × 10^−2^	5.00 × 10^−2^	3.68 × 10^−2^	1.67 × 10^−1^

**Table 5:** Std, avg, and avgtime for 10 dimensions on the CEC2017 benchmark (F21–F30).

Func.	Type	EALA	ALA	ACO	GA	DE	FATA	HHO	GJO	GWO	ABC
F21	std	5.92 × 10^1^	5.36 × 10^1^	8.93	3.91 × 10^1^	3.99 × 10^1^	4.77 × 10^1^	6.95 × 10^1^	2.39 × 10^1^	8.03	2.88 × 10^1^
	avg	2.26 × 10^3^	2.23 × 10^3^	2.33 × 10^3^	2.39 × 10^3^	2.30 × 10^3^	2.33 × 10^3^	2.30 × 10^3^	2.32 × 10^3^	2.31 × 10^3^	2.30 × 10^3^
	avgtime	1.19 × 10^−3^	1.14 × 10^−3^	1.87 × 10^−1^	6.73 × 10^−2^	1.16 × 10^−1^	4.03 × 10^−2^	7.64 × 10^−2^	5.76 × 10^−2^	3.94 × 10^−2^	1.74 × 10^−1^
F22	std	2.25 × 10^1^	1.20 × 10^1^	7.55 × 10^2^	3.50 × 10^2^	3.30	4.89 × 10^1^	3.70 × 10^2^	2.11 × 10^2^	3.09 × 10^2^	1.12
	avg	2.29 × 10^3^	2.30 × 10^3^	2.79 × 10^3^	2.61 × 10^3^	2.30 × 10^3^	2.44 × 10^3^	2.42 × 10^3^	2.41 × 10^3^	2.39 × 10^3^	2.30 × 10^3^
	avgtime	1.55 × 10^−3^	1.37 × 10^−3^	1.96 × 10^−1^	7.50 × 10^−2^	1.24 × 10^−1^	4.59 × 10^−2^	9.14 × 10^−2^	6.06 × 10^−2^	4.57 × 10^−2^	1.94 × 10^−1^
F23	std	6.97	8.54	5.59	2.11 × 10^1^	3.61	8.65	3.44 × 10^1^	1.49 × 10^1^	1.20 × 10^1^	5.85
	avg	2.61 × 10^3^	2.62 × 10^3^	2.64 × 10^3^	2.70 × 10^3^	2.62 × 10^3^	2.66 × 10^3^	2.68 × 10^3^	2.64 × 10^3^	2.62 × 10^3^	2.63 × 10^3^
	avgtime	1.48 × 10^−3^	1.61 × 10^−3^	1.66 × 10^−1^	6.03 × 10^−2^	1.21 × 10^−1^	4.71 × 10^−2^	9.56 × 10^−2^	6.16 × 10^−2^	4.78 × 10^−2^	1.97 × 10^−1^
F24	std	7.82	8.87	5.56	5.61 × 10^1^	2.39 × 10^1^	6.80 × 10^1^	9.01 × 10^1^	4.86 × 10^1^	4.05 × 10^1^	2.47 × 10^1^
	avg	2.74 × 10^3^	2.75 × 10^3^	2.77 × 10^3^	2.87 × 10^3^	2.75 × 10^3^	2.78 × 10^3^	2.81 × 10^3^	2.76 × 10^3^	2.74 × 10^3^	2.75 × 10^3^
	avgtime	1.93 × 10^−3^	1.38 × 10^−3^	1.98 × 10^−1^	7.56 × 10^−2^	1.24 × 10^−1^	5.00 × 10^−2^	9.98 × 10^−2^	6.61 × 10^−2^	5.00 × 10^−2^	1.94 × 10^−1^
F25	std	2.34 × 10^1^	2.87 × 10^1^	1.46 × 10^1^	7.97 × 10^1^	1.08 × 10^1^	2.44 × 10^1^	2.33 × 10^1^	3.98 × 10^1^	1.42 × 10^1^	7.81
	avg	2.92 × 10^3^	2.93 × 10^3^	2.94 × 10^3^	3.05 × 10^3^	2.93 × 10^3^	2.93 × 10^3^	2.94 × 10^3^	2.95 × 10^3^	2.93 × 10^3^	2.94 × 10^3^
	avgtime	1.57 × 10^−3^	1.26 × 10^−3^	1.84 × 10^−1^	6.78 × 10^−2^	1.19 × 10^−1^	4.36 × 10^−2^	9.26 × 10^−2^	5.69 × 10^−2^	4.16 × 10^−2^	1.81 × 10^−1^
F26	std	3.06 × 10^2^	3.05 × 10^2^	1.64 × 10^2^	5.49 × 10^2^	4.53 × 10^1^	1.04 × 10^2^	4.92 × 10^2^	2.48 × 10^2^	3.79 × 10^2^	4.37 × 10^1^
	avg	3.01 × 10^3^	3.04 × 10^3^	3.36 × 10^3^	3.90 × 10^3^	3.04 × 10^3^	3.14 × 10^3^	3.70 × 10^3^	3.20 × 10^3^	3.14 × 10^3^	2.99 × 10^3^
	avgtime	2.07 × 10^−3^	2.00 × 10^−3^	2.03 × 10^−1^	8.22 × 10^−2^	1.28 × 10^−1^	5.28 × 10^−2^	1.06 × 10^−1^	6.82 × 10^−2^	5.39 × 10^−2^	1.97 × 10^−1^
F27	std	1.31 × 10^1^	2.82	2.44 × 10^1^	4.90 × 10^1^	1.91	3.19 × 10^1^	6.75 × 10^1^	2.14 × 10^1^	1.16 × 10^1^	1.45
	avg	3.09 × 10^3^	3.09 × 10^3^	3.18 × 10^3^	3.22 × 10^3^	3.09 × 10^3^	3.12 × 10^3^	3.20 × 10^3^	3.11 × 10^3^	3.10 × 10^3^	3.09 × 10^3^
	avgtime	1.77 × 10^−3^	1.75 × 10^−3^	1.73 × 10^−1^	7.04 × 10^−2^	1.33 × 10^−1^	5.40 × 10^−2^	1.13 × 10^−1^	6.84 × 10^−2^	5.40 × 10^−2^	2.05 × 10^−1^
F28	std	1.70 × 10^2^	1.73 × 10^2^	9.11	2.10 × 10^2^	5.82 × 10^1^	2.01 × 10^1^	1.18 × 10^2^	1.26 × 10^2^	8.14 × 10^1^	4.49 × 10^1^
	avg	3.31 × 10^3^	3.35 × 10^3^	3.29 × 10^3^	3.54 × 10^3^	3.35 × 10^3^	3.41 × 10^3^	3.45 × 10^3^	3.42 × 10^3^	3.40 × 10^3^	3.29 × 10^3^
	avgtime	1.53 × 10^−3^	1.57 × 10^−3^	1.90 × 10^−1^	7.71 × 10^−2^	1.27 × 10^−1^	4.98 × 10^−2^	9.80 × 10^−2^	6.31 × 10^−2^	4.94 × 10^−2^	1.95 × 10^−1^
F29	std	4.01 × 10^1^	2.95 × 10^1^	7.43 × 10^1^	1.00 × 10^2^	1.86 × 10^1^	4.48 × 10^1^	1.14 × 10^2^	6.52 × 10^1^	5.46 × 10^1^	2.55 × 10^1^
	avg	3.18 × 10^3^	3.18 × 10^3^	3.35 × 10^3^	3.35 × 10^3^	3.20 × 10^3^	3.27 × 10^3^	3.42 × 10^3^	3.23 × 10^3^	3.21 × 10^3^	3.25 × 10^3^
	avgtime	1.39 × 10^−3^	1.28 × 10^−3^	1.88 × 10^−1^	7.37 × 10^−2^	1.29 × 10^−1^	4.84 × 10^−2^	9.34 × 10^−2^	6.34 × 10^−2^	4.49 × 10^−2^	1.94 × 10^−1^
F30	std	9.58 × 10^5^	8.39 × 10^5^	1.22 × 10^4^	2.25 × 10^6^	1.97 × 10^5^	6.66 × 10^5^	2.13 × 10^6^	3.72 × 10^6^	1.20 × 10^6^	5.59 × 10^5^
	avg	6.48 × 10^5^	7.49 × 10^5^	1.81 × 10^4^	3.29 × 10^6^	1.97 × 10^5^	7.88 × 10^5^	2.19 × 10^6^	1.96 × 10^6^	7.64 × 10^5^	8.46 × 10^5^
	avgtime	3.70 × 10^−3^	3.08 × 10^−3^	2.67 × 10^−1^	1.25 × 10^−1^	1.72 × 10^−1^	9.67 × 10^−2^	2.15 × 10^−1^	1.11 × 10^−1^	9.68 × 10^−2^	2.94 × 10^−1^

**Table 6:** The results of the Wilcoxon rank-sum test for 10 dimensions on the CEC2017 benchmark.

Func.	ALA	ACO	GA	DE	FATA	HHO	GJO	GWO	ABC
F1	5.01 × 10^−1^	6.97 × 10^−3^	3.02 × 10^−11^	3.02 × 10^−11^	3.02 × 10^−11^	3.02 × 10^−11^	3.02 × 10^−11^	3.02 × 10^−11^	3.02 × 10^−11^
F3	3.02 × 10^−11^	3.02 × 10^−11^	3.02 × 10^−11^	3.02 × 10^−11^	3.02 × 10^−11^	3.02 × 10^−11^	3.02 × 10^−11^	3.02 × 10^−11^	3.02 × 10^−11^
F4	2.00 × 10^−6^	5.57 × 10^−10^	8.15 × 10^−11^	5.57 × 10^−10^	2.61 × 10^−10^	3.32 × 10^−6^	2.61 × 10^−10^	2.92 × 10^−9^	5.57 × 10^−10^
F5	2.24 × 10^−2^	6.72 × 10^−10^	3.02 × 10^−11^	1.17 × 10^−4^	3.02 × 10^−11^	8.15 × 10^−11^	9.53 × 10^−7^	7.48 × 10^−2^	2.37 × 10^−10^
F6	1.05 × 10^−1^	3.02 × 10^−11^	3.02 × 10^−11^	3.02 × 10^−11^	3.02 × 10^−11^	3.02 × 10^−11^	3.02 × 10^−11^	2.44 × 10^−9^	1.75 × 10^−5^
F7	8.68 × 10^−3^	6.70 × 10^−11^	3.02 × 10^−11^	5.61 × 10^−5^	3.02 × 10^−11^	3.02 × 10^−11^	1.86 × 10^−9^	3.04 × 10^−1^	1.78 × 10^−10^
F8	1.58 × 10^−1^	1.17 × 10^−9^	3.02 × 10^−11^	3.56 × 10^−4^	1.17 × 10^−9^	5.46 × 10^−6^	1.49 × 10^−6^	8.88 × 10^−1^	6.12 × 10^−10^
F9	2.24 × 10^−2^	3.02 × 10^−11^	3.02 × 10^−11^	7.39 × 10^−1^	3.02 × 10^−11^	3.02 × 10^−11^	3.02 × 10^−11^	1.16 × 10^−7^	1.11 × 10^−4^
F10	2.23 × 10^−1^	3.02 × 10^−11^	3.35 × 10^−8^	3.03 × 10^−2^	4.98 × 10^−11^	8.31 × 10^−3^	7.98 × 10^−2^	3.92 × 10^−2^	2.67 × 10^−9^
F11	1.56 × 10^−2^	5.86 × 10^−6^	4.98 × 10^−11^	8.29 × 10^−6^	5.57 × 10^−10^	8.89 × 10^−10^	5.07 × 10^−10^	9.76 × 10^−10^	1.16 × 10^−7^
F12	1.61 × 10^−6^	3.02 × 10^−11^	4.50 × 10^−11^	3.02 × 10^−11^	3.02 × 10^−11^	4.08 × 10^−11^	4.08 × 10^−11^	1.61 × 10^−10^	3.02 × 10^−11^
F13	6.28 × 10^−6^	3.02 × 10^−11^	3.02 × 10^−11^	4.50 × 10^−11^	3.02 × 10^−11^	3.34 × 10^−11^	3.34 × 10^−11^	4.50 × 10^−11^	3.02 × 10^−11^
F14	7.96 × 10^−3^	3.02 × 10^−11^	3.02 × 10^−11^	6.70 × 10^−11^	3.02 × 10^−11^	3.02 × 10^−11^	3.02 × 10^−11^	3.02 × 10^−11^	3.02 × 10^−11^
F15	6.10 × 10^−3^	3.02 × 10^−11^	3.02 × 10^−11^	1.86 × 10^−9^	3.02 × 10^−11^	3.02 × 10^−11^	3.02 × 10^−11^	3.02 × 10^−11^	3.02 × 10^−11^
F16	6.52 × 10^−1^	7.22 × 10^−6^	7.38 × 10^−10^	5.01 × 10^−1^	4.18 × 10^−9^	6.70 × 10^−11^	1.31 × 10^−8^	1.95 × 10^−3^	6.35 × 10^−2^
F17	8.31 × 10^−3^	9.79 × 10^−5^	8.88 × 10^−6^	1.09 × 10^−5^	8.89 × 10^−10^	8.15 × 10^−5^	1.11 × 10^−4^	2.89 × 10^−3^	7.20 × 10^−5^
F18	5.07 × 10^−10^	3.02 × 10^−11^	3.69 × 10^−11^	3.02 × 10^−11^	3.02 × 10^−11^	3.02 × 10^−11^	3.02 × 10^−11^	3.02 × 10^−11^	3.02 × 10^−11^
F19	2.57 × 10^−7^	3.02 × 10^−11^	3.34 × 10^−11^	3.69 × 10^−11^	3.02 × 10^−11^	3.02 × 10^−11^	3.02 × 10^−11^	3.34 × 10^−11^	3.02 × 10^−11^
F20	6.00 × 10^−1^	1.67 × 10^−1^	6.07 × 10^−11^	4.08 × 10^−11^	1.61 × 10^−10^	3.47 × 10^−10^	7.12 × 10^−9^	1.43 × 10^−5^	2.68 × 10^−4^
F21	8.77 × 10^−1^	2.44 × 10^−9^	9.76 × 10^−10^	1.25 × 10^−4^	2.83 × 10^−8^	6.36 × 10^−5^	5.53 × 10^−8^	2.68 × 10^−4^	9.88 × 10^−3^
F22	3.40 × 10^−1^	5.57 × 10^−10^	3.02 × 10^−11^	3.08 × 10^−8^	3.02 × 10^−11^	5.09 × 10^−8^	4.20 × 10^−10^	4.08 × 10^−11^	4.98 × 10^−11^
F23	6.67 × 10^−3^	3.02 × 10^−11^	3.02 × 10^−11^	6.55 × 10^−4^	3.02 × 10^−11^	3.02 × 10^−11^	3.16 × 10^−10^	9.07 × 10^−3^	2.44 × 10^−9^
F24	1.24 × 10^−3^	1.21 × 10^−10^	3.69 × 10^−11^	8.66 × 10^−5^	8.48 × 10^−9^	3.35 × 10^−8^	1.16 × 10^−7^	5.89 × 10^−1^	9.52 × 10^−4^
F25	3.03 × 10^−3^	5.19 × 10^−7^	3.02 × 10^−11^	1.62 × 10^−1^	4.03 × 10^−3^	2.25 × 10^−4^	3.50 × 10^−3^	8.66 × 10^−5^	8.12 × 10^−4^
F26	2.15 × 10^−2^	1.09 × 10^−5^	3.50 × 10^−9^	4.11 × 10^−7^	1.73 × 10^−7^	1.16 × 10^−7^	1.25 × 10^−7^	6.10 × 10^−3^	8.66 × 10^−5^
F27	2.06 × 10^−1^	6.07 × 10^−11^	3.02 × 10^−11^	1.62 × 10^−1^	1.43 × 10^−8^	1.61 × 10^−10^	6.36 × 10^−5^	6.97 × 10^−3^	1.86 × 10^−1^
F28	6.95 × 10^−1^	6.63 × 10^−1^	5.60 × 10^−5^	3.04 × 10^−1^	2.68 × 10^−6^	1.25 × 10^−5^	1.78 × 10^−4^	1.25 × 10^−4^	6.41 × 10^−1^
F29	5.30 × 10^−1^	2.37 × 10^−10^	3.82 × 10^−10^	1.44 × 10^−3^	4.69 × 10^−8^	7.39 × 10^−11^	6.91 × 10^−4^	9.07 × 10^−3^	1.36 × 10^−7^
F30	6.35 × 10^−2^	4.36 × 10^−2^	1.60 × 10^−7^	6.20 × 10^−1^	5.55 × 10^−2^	2.25 × 10^−4^	1.44 × 10^−2^	3.63 × 10^−1^	2.51 × 10^−2^

**Table 7:** Parameter analysis of UAV path planning for large-scale environments.

Alg.	L	S	R	T	CT	CF
EALA	58.94	25.24	0.00	19.00	1.35	60.0800
ALA	62.75	34.31	0.00	28.59	1.69	64.1772
ACO	111.33	129.02	1.14	158.27	55.17	128.5076
GA	141.53	165.98	0.88	205.56	3.99	160.6451
DE	103.77	106.77	0.89	128.34	55.94	117.0715
FATA	62.75	34.31	0.02	28.59	57.55	64.2852
HHO	62.75	34.31	0.00	28.59	5.66	64.1772
GJO	95.62	102.62	0.97	114.86	17.12	108.1252
PSO	60.26	26.66	0.96	20.74	3.42	65.5234
GWO	101.14	94.21	0.91	100.61	43.89	112.7898
ABC	95.68	98.77	0.96	111.82	167.72	107.9623

**Table 8:** Parameter analysis of UAV path planning in medium-scale environment.

Alg.	L	S	R	T	CT	CF
EALA	34.61	19.38	0.00	18.38	10.13	35.5344
ALA	38.36	29.50	17.71	31.52	17.95	41.7103
ACO	48.99	44.99	41.89	52.05	256.10	55.7861
GA	71.23	77.15	46.42	99.96	15.81	80.8725
DE	47.48	42.59	31.89	46.10	21.48	52.9781
FATA	45.97	35.31	20.36	40.78	24.04	50.0449
HHO	40.70	30.72	0.00	29.95	13.06	42.1994
GJO	49.14	47.83	34.06	57.78	105.71	55.4325
PSO	47.04	46.23	29.97	47.40	16.61	52.4111
GWO	50.31	50.16	30.74	55.08	23.44	56.1405
ABC	48.95	43.14	32.72	47.02	156.02	54.5710

## Data Availability

The data that support the findings of this study are available from the corresponding author upon request. There are no restrictions on data availability.
